# The genetic underpinnings of variation in ages at menarche and natural menopause among women from the multi-ethnic Population Architecture using Genomics and Epidemiology (PAGE) Study: A trans-ethnic meta-analysis

**DOI:** 10.1371/journal.pone.0200486

**Published:** 2018-07-25

**Authors:** Lindsay Fernández-Rhodes, Jennifer R. Malinowski, Yujie Wang, Ran Tao, Nathan Pankratz, Janina M. Jeff, Sachiko Yoneyama, Cara L. Carty, V. Wendy Setiawan, Loic Le Marchand, Christopher Haiman, Steven Corbett, Ellen Demerath, Gerardo Heiss, Myron Gross, Petra Buzkova, Dana C. Crawford, Steven C. Hunt, D. C. Rao, Karen Schwander, Aravinda Chakravarti, Omri Gottesman, Noura S. Abul-Husn, Erwin P. Bottinger, Ruth J. F. Loos, Leslie J. Raffel, Jie Yao, Xiuqing Guo, Suzette J. Bielinski, Jerome I. Rotter, Dhananjay Vaidya, Yii-Der Ida Chen, Sheila F. Castañeda, Martha Daviglus, Robert Kaplan, Gregory A. Talavera, Kelli K. Ryckman, Ulrike Peters, Jose Luis Ambite, Steven Buyske, Lucia Hindorff, Charles Kooperberg, Tara Matise, Nora Franceschini, Kari E. North

**Affiliations:** 1 Department of Epidemiology, University of North Carolina at Chapel Hill, Chapel Hill, North Carolina, United States of America; 2 Carolina Population Center, University of North Carolina at Chapel Hill, Chapel Hill, North Carolina, United States of America; 3 Write InSciTe, LLC, Hebron, Connecticut, United States of America; 4 Department of Biostatistics, Vanderbilt University Medical Center, Nashville, Tennessee, United States of America; 5 Department of Laboratory Medicine and Pathology, University of Minnesota, Minneapolis, Minnesota, United States of America; 6 Genotyping Arrays Division, Illumina, Inc., San Diego, California, United States of America; 7 Division of Public Health Sciences, Fred Hutchinson Cancer Research Center, Seattle, Washington, United States of America; 8 Department of Preventive Medicine, Norris Comprehensive Cancer Center, Keck School of Medicine, University of Southern California, Los Angeles, California, United States of America; 9 Epidemiology Program, University of Hawaii Cancer Center, Honolulu, Hawaii, United States of America; 10 Kansas Health Institute, Topeka, Kansas, United States of America; 11 Division of Epidemiology & Community Health, University of Minnesota, Minneapolis, Minnesota, United States of America; 12 Department of Biostatistics, School of Public Health, University of Washington, Seattle, Washington, United States of America; 13 Institute for Computational Biology, Department of Epidemiology and Biostatistics, Case Western Reserve University, Cleveland, Ohio, United States of America; 14 Department of Genetic Medicine, Weill Cornell Medical College in Qatar, Doha, Qatar; 15 Division of Biostatistics, Washington University in St. Louis, St. Louis, Michigan, United States of America; 16 Center for Complex Disease Genomics, McKusick-Nathans Institute of Genetic Medicine, Johns Hopkins University, Baltimore, Maryland, United States of America; 17 Division of General Internal Medicine, Icahn School of Medicine at Mount Sinai, New York, New York, United States of America; 18 The Charles Bronfman Institute for Personalized Medicine, Icahn School of Medicine at Mount Sinai, New York, New York, United States of America; 19 Division of Genetic and Genomic Medicine, University of California—Irvine, Irvine, California, United States of America; 20 Institute for Translational Genomics and Population Sciences, Los Angeles Biomedical Research Institute and Department of Pediatrics at Harbor-UCLA Medical Center, Torrance, California, United States of America; 21 College of Medicine, Mayo Clinic, Rochester, Minnesota, United States of America; 22 Department of Medicine, Johns Hopkins University, Baltimore, Maryland, United States of America; 23 South Bay Latino Research Center, Graduate School of Public Health, San Diego State University, San Diego, California, United States of America; 24 Institute of Minority Health Research, University of Illinois at Chicago, Chicago, Illinois, United States of America; 25 Department of Epidemiology and Population Health, Albert Einstein College of Medicine, Bronx, New York, United States of America; 26 Departments of Epidemiology and Pediatrics, University of Iowa, Iowa City, Iowa, United States of America; 27 Information Sciences Institute, University of Southern California, Marina del Rey, California, United States of America; 28 Department of Genetics, Rutgers University, Piscataway, New Jersey, United States of America; 29 Division of Genomic Medicine, National Human Genome Research Institute, National Institutes of Health, Bethesda, Maryland, United States of America; University of Texas Health Science Center at San Antonio, UNITED STATES

## Abstract

Current knowledge of the genetic architecture of key reproductive events across the female life course is largely based on association studies of European descent women. The relevance of known loci for age at menarche (AAM) and age at natural menopause (ANM) in diverse populations remains unclear. We investigated 32 AAM and 14 ANM previously-identified loci and sought to identify novel loci in a trans-ethnic array-wide study of 196,483 SNPs on the MetaboChip (Illumina, Inc.). A total of 45,364 women of diverse ancestries (African, Hispanic/Latina, Asian American and American Indian/Alaskan Native) in the Population Architecture using Genomics and Epidemiology (PAGE) Study were included in cross-sectional analyses of AAM and ANM. Within each study we conducted a linear regression of SNP associations with self-reported or medical record-derived AAM or ANM (in years), adjusting for birth year, population stratification, and center/region, as appropriate, and meta-analyzed results across studies using multiple meta-analytic techniques. For both AAM and ANM, we observed more directionally consistent associations with the previously reported risk alleles than expected by chance (p-values_binomial_≤0.01). Eight densely genotyped reproductive loci generalized significantly to at least one non-European population. We identified one trans-ethnic array-wide SNP association with AAM and two significant associations with ANM, which have not been described previously. Additionally, we observed evidence of independent secondary signals at three of six AAM trans-ethnic loci. Our findings support the transferability of reproductive trait loci discovered in European women to women of other race/ethnicities and indicate the presence of additional trans-ethnic associations both at both novel and established loci. These findings suggest the benefit of including diverse populations in future studies of the genetic architecture of female growth and development.

## Introduction

Age at menarche (AAM) and age at natural menopause (ANM) are important events in the reproductive lifespan of a woman. Menarche, the initiation of the female menstrual cycle, occurs at 12 years on average [1,2]. In the United States (US), mean AAM is lower for African and Mexican American women, and higher for non-Hispanic women of European descent [2,3]. Yet, epidemiologic data on the average AAM of Asian American, Native Hawaiian and American Indian/Alaskan Native women are generally lacking. An earlier age at menarche has been associated with early life obesity and risk for a variety of diseases including breast and endometrial cancer, diabetes, and coronary heart disease [4–6].

Menopause, the cessation of the menstrual cycle that signifies the end of the reproductive lifespan, occurs at 51 years on average, with the majority of women experiencing a natural onset of menopause (not surgically or drug-induced) sometime between ages 45–55 years [7]. Similar to AAM, race/ethnicity appears to be an independent predictor of ANM in the US, with African and Mexican American women having earlier ANM, as compared to non-Hispanic women of European and Japanese descent [8,9]. Epidemiologic investigations of ANM in other US racial/ethnic groups are still needed. Earlier ANM is influenced by smoking status and can confer increased risk for cardiovascular disease and osteoporosis later in life, while later ANM can increase the risk of hormone-related female cancers, such as breast and endometrial cancers [10,11].

For both AAM and ANM, population-level changes have been observed in the US over the last century, wherein the average AAM decreased [1] and the average ANM has increased [12]. These trends may reflect the population-level shifts in the race/ethnicities of females living currently in the US, secular trends in obesity or smoking prevalence, or other environmental conditions supportive of a longer average female reproductive lifespan.

Given the racial/ethnic differences in AAM and ANM in the US, there remains significant interest in identifying the genetic factors that influence the timing of these reproductive events in diverse populations. Numerous candidate gene and genome-wide association studies (GWAS) have been performed for AAM and ANM, and as a result, more than 360 and 40 loci have been associated with AAM and ANM, respectively [13–23]. Although the vast majority of these studies have included only women of European descent in their discovery and validation samples, more recent GWAS have begun to include women of African (up to ~18,000 women) and East Asian ancestry (up to ~16,000 women), but have not discovered any additional loci [24–28]. Recent generalizability studies have also begun to include these populations as well as Hispanic/Latina, Native Hawaiian, and American Indian/Alaskan Native descent women [29–31] to more fully describe the transferability and allele frequency heterogeneity of these established AAM and ANM loci, as well as to discover novel race/ethnic-specific loci.

Recently developed methods for trans-ethnic meta-analysis now allow researchers to combine several populations, while accounting for heterogeneity between racial/ethnic groups [32,33]. Previous genetic epidemiologic research indicates that trans-ethnic meta-analyses improve the power to discover variants of low and moderate effect sizes and may reveal allelic heterogeneity at known genetic loci [17,26,27]. Additionally, trans-ethnic approaches may help narrow the interval of interest around loci discovered in European-descent populations. The Population Architecture using Genomics and Epidemiology (PAGE) Study, a consortium of ancestrally diverse genetic studies from the US, is well-positioned to investigate the genetics of complex traits within a trans-ethnic context [34].

Herein, we sought to analyze the roughly 200,000 SNPs genotyped on the MetaboChip (Illumina, Inc., San Diego, CA, USA), a high-density genotyping array of primarily cardiometabolic loci [35], for association with reproductive milestones in the ancestrally diverse study participants of the PAGE Study [34]. Given the known overlap between the genetic underpinnings of AAM, and related cardiometabolic traits [22], the MetaboChip provides a densely genotyped resource to search for novel reproductive associations and broadly investigate the overlap of cardiometabolic and reproductive traits. Using race/ethnicity-stratified meta-analyses (20,398 African American, 15,856 Hispanic/Latina, 8,572 Asian American, and 538 American Indian/Alaskan Native women) and a trans-ethnic modified random-effects meta-analysis of up to 42,826 ancestrally diverse women, we sought to (i) establish how many index AAM and ANM SNPs, previously described in European-descent populations, also generalize to diverse racial/ethnic groups of women in the PAGE Study, (ii) their trans-ethnic localization, and (iii) to identify novel AAM or ANM associations on the MetaboChip.

## Materials and methods

### Study participants and phenotyping

The PAGE Study was designed to generalize and estimate common genetic effects across multiple ancestral populations [34]. Briefly, the first phase of the PAGE study was comprised of a coordinating center, four large study sites/consortia [Causal Variants Across the Life Course (CALiCo) Consortium, including the Atherosclerosis Risk in Communities (ARIC) Study, Coronary Artery Risk Development in Young Adults (CARDIA), the Hispanic Community Health Study/Study of Latinos (HCHS/SOL); Epidemiologic Architecture for Genes Linked to Environment (EAGLE)-accessing the Vanderbilt University Medical Center’s biorepository (BioVU); Multiethnic Cohort (MEC); the Women’s Health Initiative (WHI)], and additional collaborating studies [The Hypertension Genetic Epidemiology Network (HyperGEN) Study, the MEC-Slim Initiative in Genomic Medicine for the Americas Type 2 Diabetes Consortium (MEC-SIGMA), Multi-Ethnic Study of Atherosclerosis (MESA), and Mount Sinai School of Medicine BioBank (BioME)]. We provide a detailed description of each study included in this analysis in our S1 Text. The datasets generated as part of the PAGE study can be accessed through the dbGaP repository (http://www.ncbi.nlm.nih.gov/projects/gap/cgi-bin/study.cgi?study_id=phs000356). All studies in this analysis obtained Institutional Review Board approval and written informed consent from all participants, with the exception of EAGLE BioVU, which obtained Institutional Review Board approval to follow an opt-out consent process, described in detail separately [36,37].

Self-reported AAM (onset of first menses) and ANM (cessation of regular menses) in years were collected by questionnaire or via medical record [38]. AAM and ANM were harmonized across the studies, as reported previously [30]. Detailed descriptions of the pseudo-continuous coding and outlier exclusions are provided in the S2 Text.

### Genotyping and imputation

The custom Illumina, Inc. iSELECT array, MetaboChip, genotyped 196,483 autosomal SNPs including the high-density genotyping of 257 regions associated with cardiometabolic traits as of 2009 [35]. As described in S2 Text, for three studies MetaboChip SNPs were imputed [MEC SIGMA, BioME, WHI African Americans [39]]. Additionally, we excluded SNPs with low minor allele frequencies (MAF), <0.1%, or that had deviations from Hardy-Weinberg Equilibrium (HWE), p-value<1x10^-6^. Additional information on the specific implementation of HWE filtering and other SNP-level quality control procedures is provided in the S2 Text.

Forty-six index SNPs had been previously associated with either AAM or ANM (or if unavailable on the MetaboChip, a proxy SNP r^2^≥0.8 in 1000 Genomes CEU sample) and represented distinct genetic loci (r^2^<0.2) (S1 Table). These SNPs included all two of the known AAM and five of the known ANM loci as of when the MetaboChip was designed, including the two strongest and most widely-generalizable AAM and ANM signals to date (*LIN28B* and *MCM8*) [22,23]. Additionally, seven of the previously-associated SNPs were located within six densely-genotyped loci (S2 Table) that were associated with AAM or ANM after the initial design of the MetaboChip.

S2 Text provides additional information on the following person-level exclusions. Briefly, we identified and excluded individuals with high inbreeding coefficients, *F >* 0.15 [40], and either excluded one woman of each 1st degree relative pair [41], or modeled relatedness using generalized estimating equations [42] and linear mixed models [43]. We generated principal components using Eigensoft for each study [44,45] and excluded ancestral outliers [46]. We excluded samples with phenotype-genotype sex discordance, low person-level call rate (<95%), or excessive heterozygosity.

Only study- and race/ethnic-specific sample sizes of 50 women or more were carried forward to statistical analyses and summarized descriptively in S3 and S4 Tables. Collectively, the studies represent 20,398 African American, 15,856 Hispanic/Latina, 8,572 Asian American, and 538 American Indian/Alaskan Native women who provided informed consent (or in the case of BioVU, did not opt out from the approved research), and had complete information on genetics, reproductive phenotypes and covariates.

### Statistical modeling and analyses

Within each study the MetaboChip SNP and reproductive trait associations were modeled under an additive genetic model and adjusted for birth year, principal components, or if applicable, also center and Type 2 Diabetes case/control status. Within each racial/ethnic group we then implemented fixed-effect inverse-variance weighted meta-analyses using METAL (version from 2011-03-25) [47], to estimate race/ethnic-specific effects for those SNPs that were informed by more than half of the maximum race/ethnic sample (n = 123,493–157,710). Additional information on our data visualization and post-hoc power analyses are provided in the S1 Text.

Array-wide significance for novel SNPs was defined as a Bonferroni p-value<2.5x10^-7^ to account for the total number of autosomal SNPs on the MetaboChip (n = 196,483). We concluded that the observed association was directionally consistent, if the trait-decreasing allele of the trans-ethnic analysis was the same as the trait-decreasing allele of previous report(s). Furthermore, using a binomial distribution we tested (p-values _binomial_ <0.05) if we observed more directional consistency than we would expect by chance (i.e. assuming that 50% of all tests would be consistent by chance alone). Generalization of previously described reproductive loci at index SNPs (or their proxies) to our samples was declared if 1) our estimate was directionally consistent with the previous reports, and 2) the SNP association had a p-value<0.0016 for AAM or p-value<0.0036 for ANM, corresponding to a Bonferroni correction for the number of independent AAM (n = 32) and ANM (n = 14) loci tested. SNPs located within densely genotyped reproductive loci, which were not index SNPs or their proxies, were considered to be significant if their association was less than a p-value Bonferroni-corrected for the number of independent signals within the given locus (independent signals pruned to r^2^<0.2 in ARIC African Americans), resulting in p-value thresholds ranging from 9.0x10^-5^ to 2.8x10^-4^ (S4 Table). Within each densely genotyped locus, statistically significant race/ethnic-specific lead SNPs (i.e. those with the lowest p-values in the locus) were considered to be potentially independent of the index SNP and warranting of additional conditional analyses, if they were in moderate to low linkage disequilibrium, LD (r^2^<0.5 in 1000 Genomes CEU sample).

At each of the densely genotyped reproductive loci, publicly available reference samples from 1000 Genomes were utilized to estimate the number of SNPs (and their base pair locations) in the European (CEU), African (YRI), Hispanic/Latino (MXL, PUR, CLM), and Asian (JPT) reference populations that are in high LD (r^2^≥0.8) with the previously reported AAM and ANM index SNPs. The percentage reduction in the putative interval of interest was then calculated by contrasting the populations with the smallest and largest LD blocks associated with these index SNPs (S5 Table).

### Modified random-effects meta-analysis

Trans-ethnic meta-analyses of AAM and ANM were conducted using a modified random-effects meta-analysis of study/race-ethnic-specific results, as implemented in Metasoft by Han and Eskin, which applies a likelihood ratio test to allow the existence of heterogeneity to be dependent on the hypothesis of association—either the alternative (random-effects) or the null hypotheses (a fixed null effect) [48]. We excluded American Indian/Alaskan Native women from the trans-ethnic meta-analyses, due to their relatively small sample size as compared to the combined trans-ethnic sample of the other racial/ethnic groups (1% for both AAM and ANM). For SNP-associations with more than half of the maximum trans-ethnic sample size for the specific trait, we estimated modified random-effects up to 22 AAM and 23 ANM study subsamples of African, Hispanic/Latina and Asian ancestry.

#### Secondary signal analysis

Next, we tested for the presence of statistically significant secondary signals using an approximate conditional method in Genome-wide Complex Trait Analysis (GCTA, version 64) [49,50] and using the same trans-ethnic reference samples as above to estimate trans-ethnic LD patterns. Adjusting for the significant lead trans-ethnic SNP at each locus, we contrasted the unconditional and approximate conditional p-values of the SNPs within the region. If an unconditional SNP association was suggestive (p-value<0.05) and not heterogeneous across race/ethnic groups in the trans-ethnic modified random-effect analysis, but became array-wide or Bonferroni-significant after adjusting for the lead SNP in the region, we concluded that this was evidence for a secondary signal in the region. This approach was repeated until no additional significant conditional SNP associations arose.

## Results

### The epidemiology of ages at menarche and natural menopause

Our final analytic samples were comprised of 44,367 and 17,100 women with AAM and ANM information from four broad racial/ethnic groups (Table 1). The biobank studies (EAGLE BioVU, BioME) and HCHS/SOL represented a wide range of ages (S3 and S4 Tables). The median age was lower and the median birth year more recent in the AAM samples, than in the ANM samples. In both the AAM and ANM analytic samples, the obesity prevalence at examination was the highest in African American women and lowest in the East Asian women (47 versus 10% weighted prevalence). MEC Native Hawaiian, Hispanic/Latina, and WHI American Indian/Alaskan Native women had intermediate obesity prevalence estimates (37–45%). In the ANM analysis samples, the prevalence of current cigarette smoking at examination was the highest in the Native Hawaiian (20%) and African American women and lowest in other Asian samples of women (14% versus 6% weighted prevalence). American Indian/Alaskan Native and Hispanic/Latina women had intermediate prevalence estimates of smoking (10–11%).

**Table 1:** Descriptive statistics for the age at menarche (AAM, n = 44,367) and natural menopause (ANM, n = 17,100) analytic samples.

**Study**		**Sample Size**	**Median Age at report (years)**	**Median Birth Year**	Median* **AAM or ANM (years)**	**Obesity (%)**	
**MENARCHE**							
AfA	ARIC	2,056	53	1935	13	48	
	EAGLE BioVU	656	41	1972	12	51	
	CARDIA	990	25	1960	12	21	
	MEC	4,410	59	1933	-	39	
	BioME**	373	47	1966	12	48	
	WHI**	11,724	61	1935	-	51	
H/L	HCHS/SOL	7,027	48	1962	13	45	
	MEC	859	59	1934	-	32	
	SIGMA-Diab**	910	59	1935	-	49	
	SIGMA-Cont**	910	59	1934	-	17	
	BioME**	512	49	1964	12	45	
	WHI	5,129	59	1936	-	37	
AsA	MEC-Hawaiian	1,364	54	1939	-	37	
	MEC-Japanese	3,725	59	1935	-	10	
	WHI	3,184	63	1933	-	12	
AI/AN	WHI	538	61	1935	-	45	
**Study**		**Sample Size**	**Median Age at report (years)**	**Median Birth Year**	Median* **AAM or ANM (years)**	**Obesity (%)**	**Current Smoking (%)**
**NATURAL MENOPAUSE**							
AfA	ARIC	569	56	1931	49	49	25
	CARDIA	150	54	1956	49	63	18
	HyperGen	189	57	1940	47	63	20
	MEC	1,598	62	1930	-	38	16
	MESA	583	65	1936	49	52	17
	WHI**	4,209	62	1934	50	50	12
H/L	HCHS/SOL	1,940	58	1952	49	49	16
	MEC	416	60	1934	-	31	12
	SIGMA-Diab**	225	60	1933	-	31	9
	SIGMA-Cont**	61	61	1932	-	20	8
	MESA	509	63	1938	49	51	12
	BioME**	93	55	1958	50	55	-
	WHI	2,027	60	1936	50	36	6
AsA	MEC-Hawaiian	567	57	1936	-	33	20
	MEC-Japanese	1,822	61	1932	-	7	8
	MESA	299	65	1936	50	5	2
	WHI	1,659	63	1932	50	10	4
AI/AN	WHI	184	61	1935	50	41	10

*Median estimates not available for MEC or WHI for AAM, or MEC for ANM, due to their categorical ascertainment of these traits (see S1 and S2 Text for more information on the pseudo-continuous recoding of these traits).

**Studies which include imputed MetaboChip SNP data.

Abbreviations: AfA = African American, AI/AN = American Indian/Alaskan Native, AsA = Asian American, Cont = Type 2 Diabetes Controls, Diab = Type 2 Diabetes Cases, H/L = Hispanic/Latina

### Generalization of previously reported reproductive trait associations

In our trans-ethnic AAM analyses in women of African, Hispanic/Latina and Asian descent, we generalized the association at *LIN28B* with AAM at array-wide significance (S1 Fig). Even though genotyping in the region is sparse on the MetaboChip, the strongest SNP association in the region (rs7759938) was a previously published European descent index SNP [13,17] and was directionally consistent with the previously reported risk allele (T). This SNP association was significant in the African, Hispanic/Latina, and Asian American samples after adjusting for the number of independent loci tested with AAM, and directionally consistent in American Indian/Alaskan Native women (Table 2).

**Table 2:** Generalization of five previously described age at menarche and natural menopause loci to multiple race/ethnic groups or trans-ethnically.

GWAS Ref*	SNP	GWAS Decreas-ing Allele*	Previous GWAS Effect (years)*	Chr	BP	Gene(s)*	Pop	Freq	Effect (years)	SE	P-value	P-value hetero-geneity	Sam-ple Size
**MENARCHE**													
[20]	rs823114*	G	-0.03	1	205719532	*NUCKS1*, *RAB7L1*	AfA	0.754	-0.044	0.02	*0*.*024*	0.146	20199
							H/L	0.589	-0.064	0.021	*0*.*002*	0.098	15343
							AsA	0.469	-0.008	0.02	0.744	0.819	8266
							AI/AN	0.568	-0.100	0.11	0.382	-	538
							TE**	0.636	-0.042	0.02	**1.36E-03**	-	43808
[13,17]	rs7759938	T	-0.09	6	105378954	*LIN28B*	AfA	0.464	-0.080	0.02	**2.15E-06**	0.501	20206
							H/L	0.719	-0.106	0.02	**3.22E-06**	0.671	15345
							AsA	0.702	-0.171	0.03	**1.00E-10**	0.733	8269
							AI/AN	0.699	-0.302	0.12	*0*.*011*	-	538
							TE**	0.601	-0.106	0.01	**4.71E-18**	-	43820
[13,17]	rs2090409	A	-0.1	9	108967088	*TMEM38B*	AfA	-	-	-	-	-	-
							H/L	0.325	-0.087	0.02	**7.72E-05**	0.728	15342
							AsA	0.432	-0.034	0.02	0.165	0.061	8249
							AI/AN	0.324	-0.043	0.11	0.700	-	538
							TE**	0.358	-0.060	0.02	**1.35E-04**	-	28222
**NATURAL MENOPAUSE**													
[15,18]	rs11668309*	T	-0.49	19	55833460	*BRSK1*, *TMEM150B*	AfA	0.238	-0.031	0.03	0.278	0.258	7294
							H/L	0.343	-0.2071	0.06	**2.52E-04**	0.892	5271
							AsA	0.145	-0.038	0.04	0.361	0.821	4348
							AI/AN	0.376	0.175	0.50	0.729	-	185
							TE**	0.241	-0.065	0.02	*0*.*011*	-	16913
[19]	rs236114	C	-0.5	20	5935385	*MCM8*	AfA	0.904	0.017	0.04	0.694	0.951	7108
							H/L	0.832	-0.215	0.07	**2.99E-03**	0.408	5270
							AsA	0.945	-0.100	0.10	0.303	0.722	4049
							AI/AN	0.870	-0.278	0.75	0.710	-	185
							TE**	0.901	-0.051	0.03	0.205	-	16427

*When the index SNP was not genotyped on the MetaboChip, the proxy SNP in tight linkage disequilibrium (r^2^> = 0.8 in 1000 Genomes pilot 1 CEU) with the lowest p-value in the African American sample was chose to represent the index signal. If more than one SNP represented the same locus (within 500kb of each other) on the MetaboChip, only the SNPs r2<0.2 in ARIC African Americans (or HCHS/SOL Hispanic/Latinos, when missing) were included in this table allowing preference for index SNPs, and in most cases SNPs from multiple citations. The decreasing (coded) allele and previous effect size for proxies were assigned assuming that the risk index SNP would have a similar allele frequency (either minor or major) and effect as the selected proxy SNP.

**Modified random-effects trans-ethnic meta-analysis across three racial/ethnic groups (African, Hispanic/Latina, and Asian Americans). American Indian/Alaskan Native samples were not included due to their relative small sample size.

Significant SNP-associations (p<0.05/32 menarche SNPs tested; p<0.05/14 menopause SNPs tested; all SNPs shown in S1 Table) shown in bold.

Nominally significant p-values (p<0.05) shown in italics.

All SNPs are oriented on positive strand and positions based on Build 37.

Abbreviations: AfA = African American, AI/AN = American Indian/Alaskan Native, AsA = Asian American, BP = Base pair, Chr = Chromosome, Freq = Frequency for coded decreasing allele, GWAS = Genome-wide association study, H/L = Hispanic/Latina, Pop = Racial/ethnic group or trans-ethnic analysis, TE = Trans-ethnic modified random effects, MA = Minor Allele, N = Sample Size, SE = Standard Error, SNP = Single nucleotide polymorphism.

In addition, we observed Bonferroni-significant evidence of generalization to diverse racial/ethnic groups at two other AAM loci. The index/proxy AAM SNPs at *NUCKS1* and *TMEM38B* were most strongly associated in the Hispanic/Latina subsample, and were also significant in the trans-ethnic meta-analysis and directionally consistent with the previously reported risk allele in all race/ethnic groups (Table 2).

In our trans-ethnic ANM analyses, we observed evidence of diverse generalization at two ANM loci after accounting for the number of independent loci tested with ANM. The proxy SNP at *BRSK1* and the selected index SNP at *MCM8* were significantly associated in Hispanic/Latinas (trait-decreasing allele frequency, TDAF, 34% and 83%; p-value<3x10^-3^; Table 2). The *BRSK1* and *MCM8* associations were not significant in the trans-ethnic meta-analysis or directionally consistent with the trait-decreasing allele across all race/ethnic groups.

The first index SNP at *MCM8*, [rs236114 [19]] was in moderate LD (1000 Genomes AMR r^2^ = 0.36) with another SNP, rs16991615 [15,18,23], which was both Bonferroni significantly associated in Hispanic/Latinas (TDAF 94%, p-value = 7.14x10^-6^) in the trans-ethnic sample (TDAF 97%, p-value = 1.91x10^-6^; S2 Fig) and associated with ANM in a directionally-consistent manner across all race/ethnic groups. Yet within the 9 Hispanic/Latina studies this SNP association exhibited evidence of effect heterogeneity (p-value_heterogeneity_ = 3.43x10^-4^), which in S3 Fig appeared to be driven by ANM-increasing effects among the MEC and MEC SIGMA Type 2 Diabetes cases, which was inconsistent with the previously reported ANM reducing allele and with the observed direction of effect in the WHI Native American/American Indian subsample. Although this apparent effect heterogeneity could be due to chance, it is also possible it could reflect differences in relevant pre-menopausal environments/health statuses of these specific MEC subsamples (e.g. gene-environment interactions). Additionally, approximate conditional analyses revealed that the significant association between rs16991615 and ANM at *MCM8* may be independent from rs236114 in our sample of Hispanic/Latinas (p-value_conditional_ = 6.2x10^-4^).

Next, across all 32 AAM and 14 ANM loci on the MetaboChip, we assessed the directional consistency between our race/ethnic-specific and trans-ethnic results and previously reported risk-associated alleles (S1 Table). The number of directionally-consistent SNP associations with AAM exceeded our expectation in all race/ethnic groups (p-values_binomial_<0.01) and trans-ethnic results (p-values_binomial_ = 1.2x10^-6^), with the exception of American Indian/Native American women (p-value_binomial_ = 0.11). For ANM the number of directionally consistent SNP-associations also exceeded our expectation based on chance in all race/ethnic (p-value_binomial_≤0.03) and trans-ethnic results (p-value_binomial_ = 0.01), with the exception of African American women (p-value_binomial_ = 0.18).

### Generalization at densely genotyped reproductive trait loci

**Fig 1:**
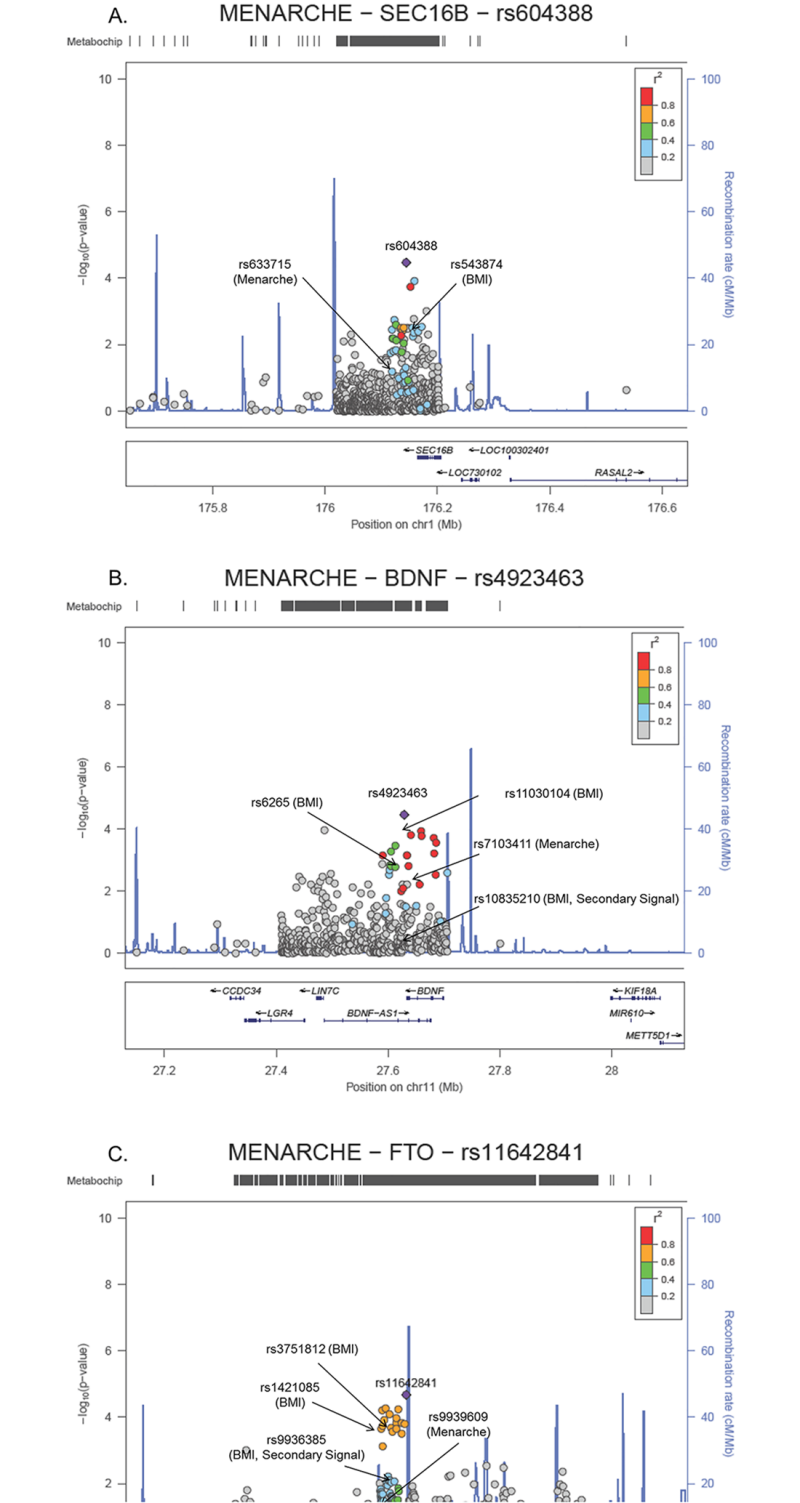
Regional plots for age at menarche Bonferroni-significant loci at *SEC16B* (Panel A), *BDNF* (Panel B) and *FTO* (Panel C), showing previously published body mass index (BMI) primary and secondary SNP associations, using a modified random-effects trans-ethnic meta-analysis of more than 31,000 women.

Three of the six densely genotyped ANM loci, *SEC16B*, *BDNF* and *FTO*, generalized to the trans-ethnic sample at a lead SNP that was in moderate LD with at least one previously reported index SNP for AAM and ANM (r^2^>0.2; Table 3 and Fig 1). At *SEC16B*, the lead Hispanic/Latina SNP (rs78368018-A; MAF 0.3%) was significant after Bonferroni correction and also in moderate LD with the index SNP (rs633715-C; trans-ethnic MAF 14.9%). However, the lead SNP had nominal evidence of effect heterogeneity across three studies of Hispanic/Latinas (p-value_heterogeneity_ = 0.03, Table 2) that was driven by a subsample of the MEC (S4 Fig). Patterns of LD at *BDNF* and *FTO* revealed that the AAM signal aligned more closely with the primary BMI signal than with other independent signals for BMI previously reported at these loci [51,52]. At four additional, albeit non-significant, densely genotyped loci, LD patterns revealed that our lead AAM or ANM SNPs were dependent on the previously reported index SNPs (S2 Table; S5 Fig).

**Table 3:** Three densely-genotyped MetaboChip loci with Bonferroni-significant associations with age at menarche across multiple race/ethnic groups or trans-ethnically.

GWAS Ref	Gene	Chr	Start/Stop BP	P-value*	Index GWAS SNP (Position)	Pop	Top MetaboChip SNP	Position	MA	Freq	Effect (years)	SE	P-value	P-value hetero-geneity	Sam-ple Size
[13]	*SEC16B*	1	177753776/177936525	2.82E-04	rs633715 (177852580)	AfA	rs75552107	177925677	G	0.158	0.127	0.04	6.85E-04	0.891	18599
						H/L	rs78368018	177777983	A	0.003	-0.825	0.21	**9.42E-05**	*0*.*038*	12787
						AsA	rs7518576	177849963	T	0.459	0.081	0.03	1.19E-03	0.885	8265
						TE**	rs604388	177877979	C	0.427	-0.060	0.01	**3.39E-05**	-	31349
[20]	*BDNF*	11	27452706/ 27749725	2.48E-04	rs7103411 (27700125)	AfA	rs113940328	27530168	C	0.053	0.140	0.04	**2.31E-04**	0.510	20209
						H/L	rs11030104	27684517	G	0.173	0.076	0.03	5.04E-03	0.851	15346
						AsA	rs1491850	27749725	C	0.412	0.073	0.02	3.38E-03	0.933	8273
						TE**	rs4923463	27672500	G	0.177	0.068	0.02	**3.54E-05**	-	43824
[13]	*FTO*	16	53539509/ 54185787	8.98E-05	rs9939609 (53820527)	AfA	rs76299885	53950231	A	0.136	-0.119	0.04	3.48E-03	0.483	19545
						H/L	rs112372930	53898024	A	0.017	0.325	0.09	5.28E-04	0.112	13527
						AsA	rs150356630	53830590	T	0.075	-0.198	0.05	1.43E-04	0.864	8273
						TE**	rs11642841	53845487	A	0.149	-0.073	0.02	**2.07E-05**	-	43797

*Bonferroni correction for the number of SNPs r2<0.2 in region in MetaboChip data from the ARIC African Americans (n = 1419 males, n = 2332 females), using a 50-SNP window and shifting the window in each iteration by 5 SNPs.

**Strongest SNP marker in modified random-effects trans-ethnic meta-analysis across three race/ethnic groups (African, Hispanic/Latina, and Asian Americans).

Significant SNP-associations below specific Bonferroni p-values for a given locus are shown in bold.

Nominally significant heterogeneity p-values (p<0.05) shown in italics.

Trans-ethnic P-value represented a modified Han and Eskin p-value.

All SNPs are oriented on positive strand and positions based on Build 37.

Abbreviations: AfA = African American, AsA = Asian American, BP = Base pair, Chr = Chromosome, Freq = Frequency for coded decreasing allele, GWAS = Genome-wide association study, H/L = Hispanic/Latina, Pop = Racial/ethnic group or trans-ethnic analysis, TE = Trans-ethnic modified random effects, MA = Minor Allele, N = Sample Size, Ref = Reference, SE = Standard Error, SNP = Single nucleotide polymorphism.

Lastly, we harnessed publicly available information on the LD blocks tagged by the index SNPs for AAM and ANM to inform narrowing of the putative interval around the loci that generalized to Hispanic/Latinas. Specifically, we found that the percent reduction in the base pair interval of interest (based on the location of SNPs in strong LD, r^2^≥0.8, with the index SNP of interest) was 77–96% across five AAM loci (*SEC16B*, *TRIM66*, *BDNF*, *GPRC5B*, *FTO*; S5 Table) or, in the case of *TMEM18* this approach pointed to one other SNP (rs7559547). For the ANM signal at *FNDC4*, the percent reduction was less dramatic (28% reduction) in the base pair interval of interest. The largest LD blocks were found in the 1000 Genomes CEU, whereas the smallest LD blocks were noted in either YRI or AMR reference populations.

### Trans-ethnic array-wide associations

**Fig 2:**
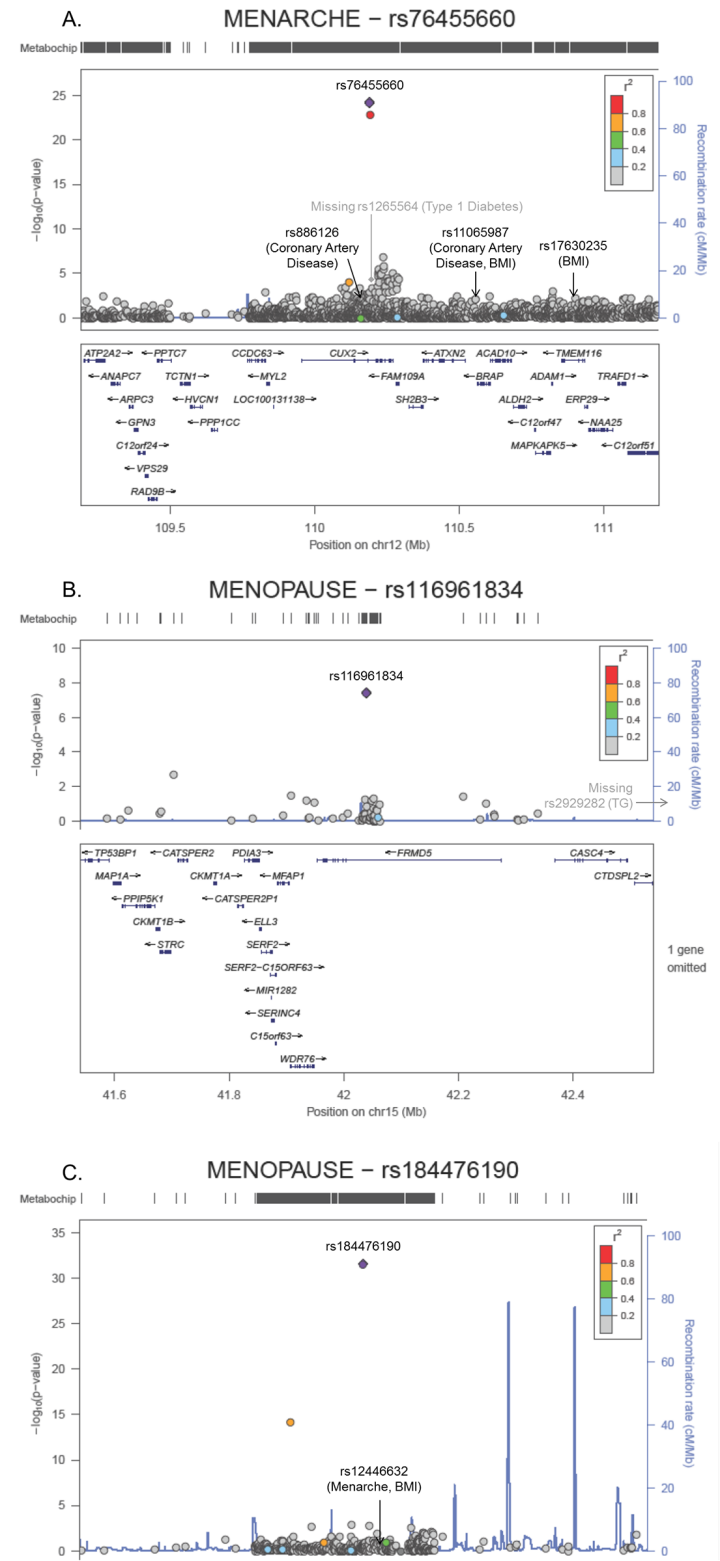
Regional plots of the novel array-wide significant age at menarche (Panel A: *CUX2*) and natural menopause loci (Panels B,C: *FRMD5*, *GPRC5B*) using a modified random-effects trans-ethnic meta-analysis of more than 31,000 women, and showing independence from previously published cardiometabolic SNP associations (shown in gray if missing).

In our trans-ethnic meta-analyses, we observed evidence of array-wide (p-value<2.5x10^-7^) novel associations with AAM at *CUX2*, and with ANM at *FRMD5* and *GPRC5B*. The lead SNPs at these three loci were all highly variable across studies but were on average low frequency SNPs (Table 4), and in weak LD with most SNPs in the region (r^2^<0.2; Fig 2A–2C). As shown in S6 Fig, the estimated effect for each novel SNP was strongest in Hispanic/Latinas than the other racial/ethnic groups, and in the case of *GPRC5B* showed evidence of heterogeneity among Hispanic/Latinas (Table 4). The lead SNPs were observed in predominantly one ancestral group, such as African (rs76455660 at *CUX2*, MAF = 1.7% 1000 Genomes AFR; rs184476190 at *GPRC5B*, MAF = 0.9% AFR) and Asian ancestries (rs116961834 at *FRMD5*, MAF = 6.6% in 1000 Genomes EAS), and several other race/ethnic samples were filtered out due to low frequency (MAF<0.1%), which yielded analytic sample sizes 61–75% of the total trans-ethnic sample for the given trait.

**Table 4:** Three loci with trans-ethnic array-wide significant modified random-effects associations* at novel age at menarche or natural menopause loci.

SNP	Chr	BP	Gene	Decreasing Allele	Pop	Freq	Freq Range	Effect* (years)	SE	P-value	P-value Het or Tau^2^	N
**MENARCHE**												
rs76455660	12	111705293	*CUX2*	C	AfA	0.837	0.806–0.991	-0.106	0.04	7.06E-03	1E-01	19435
					H/L	0.998	0.998–0.999	-0.661	0.06	**2.32E-26**	4E-01	7255
					TE	0.955	0.806–0.999	-0.108	0.12	**6.29E-25**	0.09	26690
**NATURAL MENOPAUSE**												
rs116961834	15	44252119	*FRMD5*	T	AfA	0.015	0.002–0.038	0.138	0.25	5.77E-01	5E-01	4109
					H/L	0.001	0.001–0.002	-1.390	0.20	**1.15E-11**	6E-02	4273
					AsA	0.087	0.055–0.128	-0.016	0.04	7.06E-01	7E-01	4348
					TE	0.045	0.001–0.128	-0.095	0.14	**3.74E-08**	0.13	12730
rs184476190	16	19893802	*GPRC5B*	C	AfA	0.037	0.007–0.136	-0.200	0.11	6.22E-02	4E-01	6715
					H/L	0.002	0.002–0.003	-3.081	0.27	**2.72E-30**	**1E-07**	4273
					TE	0.030	0.001–0.136	-0.494	0.44	**2.91E-32**	1.61	10988

*Strongest SNP marker in modified random-effects trans-ethnic meta-analysis informed by studies from up to three race/ethnic groups (African, Hispanic/Latina, and Asian American women). P-value represented a modified Han and Eskin p-value. Race/ethnic fixed-effect estimates and p-values of heterogeneity are shown for illustrative purposes, as the modified random-effects meta-analysis was run on studies separately.

Significant SNP-associations below specific Bonferroni p-values for a given locus are shown in bold.

All SNPs are oriented on positive strand and positions based on Build 37.

Abbreviations: AfA = African American, AsA = Asian American, BP = Base pair, Chr = Chromosome, Freq = Frequency for coded decreasing allele, Het = Heterogeneity p-value from race/ethnic specific fixed-effect meta-analysis, H/L = Hispanic/Latina, N = Sample Size, Pop = Population, SE = Standard Error, SNP = Single nucleotide polymorphism, TE = Trans-ethnic modified random effects.

#### Secondary signal analysis

As shown in S7 Fig, three AAM loci (*SEC16B*, *BDNF*, *CUX2*) had suggestive evidence of secondary signals, which were in low LD (r^2^<0.2) with the primary AAM signal observed in our unconditional trans-ethnic analyses.

At *SEC16B*, variation at the frequency of the SNP representing the possible AAM secondary signal (rs114548967-G the decreasing allele; p-value_conditional_ = 2.18x10^-4^) was driven by African and Hispanic/Latina ancestries and varied between from 0.3% to 18.6% across the 12 samples contributing to the trans-ethnic meta-analysis. In the case of *BDNF*, this Bonferroni-significant secondary signal (rs113940328-C; p-value_conditional_ = 3.90x10^-5^) was monomorphic in 1000 Genomes EUR, but varied in MAF from 0.4% to 7.4% across the 15 samples contributing to the trans-ethnic meta-analysis. This secondary SNP was independent from the previously established BMI primary and secondary signals (r^2^<0.01 with other SNPs in AFR) [51,52]. At *CUX2* the array-wide significant secondary signal (rs10849931-C; p-value_conditional_ = 1.05x10^-7^) was in weak LD with a previously described SNP for coronary artery disease (rs886126, r^2^ = 0.5 in 1000 Genomes EUR) [53] and weakly associated with the other previously described trait associations in the region (r^2^≤0.2). However, unlike the primary signal at this region (rs76455660-T, which is monomorphic in 1000 Genomes EUR) the lead conditional SNP was present in all race/ethnic groups, with remarkable variation in allele frequency across the 21 ancestry and study-specific groups analyzed jointly (9.3% to 62.3%).

## Discussion

Our trans-ethnic meta-analysis of reproductive traits has expanded our understanding of the transferability of reproductive trait loci discovered in women of European descent to other race/ethnic groups. First, we observed more directionally consistent trans-ethnic associations than we expected by chance across all 32 AAM and 14 ANM loci on the MetaboChip (p-values_binomial_ of 1.2x10^-6^ and 0.01, respectively). Second, we generalized six AAM loci (*NUCKS1*, *LIN28B TMEM38; SEC16B*, *BDNF*, *FTO*), and two ANM loci (*BRSK1*, *MCM8*) to African, Hispanic/Latina, Asian or American Indian/Alaskan Native women, observing at each locus directional consistency between our trans-ethnic risk alleles and previous reports among European descent women [13,15,17–19]. This suggests that much of the currently known genetic architecture for AAM and ANM appears to be transferable across ancestrally diverse racial/ethnic groups.

Additionally, we conducted an array-wide analysis of AAM and ANM, and identified array-wide significant SNPs at three novel loci (AAM: *CUX2*; ANM: *FRMD5*, *GPRC5B*), which were most frequent in populations with African and Asian ancestry (*CUX2*, *GPRC5B*; and *FRMD5*, respectively). Even though previous studies have associated variation in *CUX2* with Type 1 diabetes, coronary artery disease, atrial fibrillation and most recently with AAM [22,53–55], our novel signals appear to be distinct and infrequent in populations of European descent. According to HaploReg v4.1, the novel SNPs at *CUX2* and *FRMD5* are both predicted to be enhancers in the brain, which is consistent with the modulatory effect that neurotransmitter and gonadal hormones may have on each other [56]. A SNP in *FRMD5* has been previously associated with triglycerides [57], but it is 6kb downstream and in weak LD with our SNP in 1000 Genomes CHB+JPT (r^2^ = 0.01). Common genetic variation near *GPRC5B* has been previously associated with both BMI and AAM [20,51,52], but has not previously been associated with ANM. In HaploReg, the lead SNP intronic to *GPRC5B* was a predicted histone mark, promoter and/or enhancer in brain and ovary tissue, as well as having DNAase activity in ovary and several other tissues and binding affinity to neuron-restrictive silencer factor.

Our results, however, are limited by the imbalance in sample sizes available for the AAM and ANM analyses, and the relatively low proportion of established AAM and ANM loci from studies of European descent women to date available on the MetaboChip (9% and 32%, respectively). Although our exclusion of extreme values restricted our analytic sample size, it allowed us to report on the common genetic causes of normal variation in AAM and ANM. Additionally, our use of the MetaboChip as a common dense-genotyping array to all PAGE studies, and with consistent genotype calling and quality control applied to each study, is a key strength of this study. Nonetheless our ascertainment of AAM and ANM did rely primarily on self-report. Lastly, the available sample size of racial/ethnic minority women who experienced these reproductive milestones is only a fraction of the samples of European descent women published on previously [22]. Due to relative paucity of genetic data on minority women, we did not have sufficient sample sizes to achieve statistical power to identify genetic associations of low frequency variants (MAF<5%) in most of our analyses, to seek independent replication of our novel significant findings (Table 4), or to systematically explore the role of gene-environment interactions on our findings. Yet, our observation of enrichment of directional consistency (S1 Table) suggests that given sufficient power or more comprehensive genotyping arrays, additional AAM and ANM loci may be significantly associated with reproductive traits in minority women. Larger samples of diverse women are needed to investigate all currently known AAM and ANM loci, establish statistical significance and describe the magnitude of the novel genetic effects on reproductive traits with more precision.

Our findings advance our current understanding of the scope of race/ethnic groups, to which previously reported reproductive traits may be generalized, albeit often at another SNP within the previous association signal. Specifically, we were able to generalize the widely-replicated *LIN28B* association (e.g. in African, Hispanic/Latina and East Asian studies) [24,26,28,31] to a more diverse group of Asian ancestries including Native Hawaiian women from the MEC (p-value = 1.0x10^-10^), as well as to American Indian/Alaskan Native women from WHI, albeit at nominal significance (p-value = 0.01). We also extended evidence of the *NUCKS1* association with AAM beyond women of European descent to a trans-ethnic sample of women for the first time. Although all race/ethnic groups in our study had effects that were directionally consistent with previous reports at *NUCKS1*, only African American and Hispanic/Latina women were nominally associated with AAM (p-value≤0.02; Table 2).

Even though several studies have previously generalized reproductive trait associations at *TMEM38B* (AAM), *BRSK1* and *MCM8* (ANM) to African, Hispanic/Latina and East Asian ancestries [24–26,28,31], our study is the first to investigate heterogeneity within and across populations with distinct ancestries. As illustrated by our heterogeneous findings at *MCM8* (S3 Fig), the role of within group heterogeneity should be investigated in future studies of populations with European admixture, like Hispanic/Latinos [25]. Similar to previous work, we noted that *MCM8* also replicated in our sample of Hispanic/Latinas [31], even though it did not generalize to any other racial/ethnic group. This finding and the generalization of several other loci to Hispanic/Latinas may be due to their European admixture [58], or perhaps a less similar genetic architecture of reproductive traits between European and the other race/ethnic groups analyzed herein. Using the densely genotyped regions of the MetaboChip, we also demonstrated how diverse samples can help identify potential independent signals and putative variants/regions of interest for future functional follow up (S5 Table). For example, we also observed evidence that the *MCM8* region may harbor two independent signals for ANM in Hispanic/Latinas [23]. Yet, the role of ancestral differences or environmental exposures/interactions in the observed findings warrants further research [25].

Lastly, we also observed that our Bonferroni-significant AAM associations were in moderate to strong LD (r^2^>0.2; Fig 1) with the previously reported putative variants of the primary association signals at *BDNF* and *FTO* (Fig 1B and 1C). Previously, secondary signals have not been described at *SEC16B* for BMI [52], and the secondary signal we observe with AAM at *BDNF* appears to be distinct from BMI secondary signals based on our trans-ethnic LD estimates (r^2^<0.2; Fig 1A). These findings suggest that the study of AAM may yield additional insights into the genetic architecture of growth and development than studying BMI alone. The co-localization of genetic signals further supports the shared genetic underpinnings of early life growth and development in both females and males using various methodologies [59–63]. A recent study highlighted the extent of overlapping genetic loci involved in these interrelated traits, observing that the genetics of age at first birth positively correlated with the genetics of birth weight, AAM and age at voice breaking, and negatively with the genetics of smoking, BMI and ANM [64]. Even though we did not data necessary to disentangle the genetic effects on AAM and BMI in early and late life in this current PAGE Study, an increasing body of work suggests that early life growth can influence both puberty and downstream cardiometabolic consequences [22]. Future trans-ethnic research should leverage longitudinal data or casual inference methods, when attempting to further decompose the complex relationship between the genetics of growth and development across the life course.

## Conclusions

Our study is the first trans-ethnic analysis of female reproductive traits to our knowledge. Future trans-ethnic meta-analyses should include large, diverse samples with dense genotyping 1) to fine-map the reproductive trait association signals described herein, 2) to examine the joint role of functional genetic variants and environmental risk factors, and 3) to describe genetic risk factors for extreme AAM or ANM and predict their effects on the reproductive windows of women of diverse race/ethnic groups. Our findings provide support for the relevance of multiple reproductive loci to racially/ethnically diverse groups of women, and the presence of a complex genetic architecture underpinning female growth and development across the life course.

## Supporting information

S1 Text Study descriptions. (PDF)

S2 Text Supplemental methods. (PDF)

S1 Table Evidence of generalization at 46 previously described age at menarche and natural menopause signals across multiple race/ethnic groups. (PDF)

S2 Table Best marker SNPs at seven previously described age at menarche and natural menopause loci on the MetaboChip across multiple race/ethnic groups. (PDF)

S3 Table Descriptive statistics for the sample used in analysis of age at menarche. (PDF)

S4 Table Descriptive statistics for the sample used in analysis of age at natural menopause. (PDF)

S5 Table Using the set of SNPs in high LD (r^2^≥0.8) with European index SNP in African, Hispanic, and Asian American populations to narrow the region of interest. (PDF)

S1 Fig Regional plot for trans-ethnic array-wide significant association signal between *LIN28B* and AAM using a modified random-effects trans-ethnic meta-analysis of more than 43,000 women. (PDF)

S2 Fig Regional plot for trans-ethnic Bonferroni-significant association signal between *MCM8* and ANM using a modified random-effects trans-ethnic meta-analysis of more than 16,000 women. (PDF)

S3 Fig Forest plot of effect (p-value of heterogeneity = 4x10^-4^) in the fixed-effect meta-analysis (FE META) across eight study samples [Multiethnic Cohort Study = MEC, MEC-Slim Initiative in Genomic Medicine for the Americas Type 2 Diabetes Consortium = MEC SIGMA, Women’s Health Initiative = WHI, WHI American Indian/Alaskan Native = WHI AI/AN (*not included in Hispanic/Latina fixed-effect meta-analysis), Mount Sinai School of Medicine BioBank = BioME, Hispanic Community Health Study/Study of Latinos = HCHS/SOL, Multi-Ethnic Study of Atherosclerosis = MESA) of 5,258 Hispanic/Latinas at an index SNP at *MCM8* (rs16991615) with ANM. (PDF)

S4 Fig Forest plot of effect heterogeneity (p-value of heterogeneity = 0.04) across three studies (Multiethnic Cohort Study = MEC, Women’s Health Initiative = WHI, Hispanic Community Health Study/Study of Latinos = HCHS/SOL) of 12,787 Hispanic/Latinas at the best-marker fixed-effect meta-analysis (FE-META) at *SEC16B* (rs78368018) with AAM. (PDF)

S5 Fig Regional plots of non-significant modified random-effects trans-ethnic associations with dense-genotyped reproductive loci on the MetaboChip (AAM: *TMEM18*, *TRIM66*, *GPRC5B*; ANM: *FNDC4*) in up to 43,172 women with age at menarche and 16,913 women with age at natural menopause, showing the SNPs associated with body mass index (BMI) and triglycerides (TG) in previous studies, including the PAGE Study African American (AA) subsample, as well as another SNP that was associated with the other reproductive trait in this sample. (PDF)

S6 Fig Forest plots of three novel array-wide significant modified random-effect estimates (p-values<4x10^-8^; shown by the black box) and the contributing race/ethnic (African American in green; Hispanic/Latina in blue; Asian American in red) and study-specific effect estimates and their 95% confidence intervals (with double bars indicating full range not shown). (PDF)

S7 Fig Regional plots of unconditional findings (left; r^2^ based off of significant lead unconditional SNP) and approximate conditional findings after accounting for top SNPs in the region (right; r^2^ based off of unconditional lead SNP, noting significant lead conditional SNP) with age at menarche at *SEC16B*, *BDNF*, and *CUX2* in more than 31,000 women. (PDF)
