# Supplementary material for: The genetic underpinnings of variation in ages at menarche and natural menopause among women from the multi-ethnic Population Architecture using Genomics and Epidemiology (PAGE) Study: A trans-ethnic meta-analysis
Source: PLoS One. 2018 Jul 25;13(7):e0200486. doi: 10.1371/journal.pone.0200486 (PMC6059436; doi:10.1371/journal.pone.0200486)
Supplement: S1 Text — (PDF) [file pone.0200486.s001.pdf]

S1 TEXT

STUDY DESCRIPTIONS

The Population Architecture using Genomics and Epidemiology (PAGE) study has been funded to examine the epidemiologic architecture of established common genetic variants for complex human diseases by the National Human Genome Research Institute (https://www.pagestudy.org) [1]. The larger PAGE study is coordinated by a Coordinating Center and is structured into four sub-consortia, each representing one or more US racial/ethnic groups. Collectively the PAGE study includes several cohorts with information on female reproductive milestones such as the Atherosclerosis Risk in Communities Study (ARIC), the Vanderbilt University Medical Center's DNA biobank (EAGLE BioVU), the Coronary Artery Risk Disease in Young Adults study (CARDIA), the Hispanic Community Health Study/Study of Latinos (HCHS/SOL), the Multiethnic Cohort (MEC), Mount Sinai Biobank Program (BioME), and the Women's Health Initiative (WHI). Additionally, for this analysis the PAGE study also reached out to additional studies with either age at menarche (AAM) or age at natural menopause (ANM) [e.g. the Hypertension Genetic Epidemiology Network (HyperGen), the Multi-Ethnic Study of Atherosclerosis (MESA), Slim Initiative in Genomic Medicine for the Americas Type 2 Diabetes Consortium (SIGMA)] to expand the sample size. The majority of studies included in this meta-analysis were population-based studies of cardiovascular disease or cancer.

Self-reported race/ethnicity was obtained by questionnaire from each study, with the exception of the EAGLE BioVU biobank, which obtained information on observer-reported race/ethnicity (or country of birth) from the participant's medical record [2,3], and used to stratify each study by these racial/ethnic groups. The MEC-SIGMA was a Type 2 Diabetes case-control study from Los Angeles, CA and predominantly represents women of Mexican descent [4]. Similarly, more than half of the Hispanic/Latina participants from MEC, MESA, and WHI were of Mexican descent. In contrast, the Hispanic/Latina samples of the community-based HCHS/SOL

and BioME biobank represented communities and patients from several non-Mexican backgrounds, such as Central and South American, Cuban, Dominican, and Puerto Rican [5-7]. MEC Asian American participants were analyzed separately by their self-reported ancestry (Japanese or Native Hawaiian), to allow for separate estimation of covariates. The MESA Asian American samples were exclusively of Chinese descent. The WHI Asian American samples were of Chinese and Japanese descent primarily, but also represented other Asian backgrounds such as Native Hawaiian, Filipino, Korean, and Vietnamese [8]. Women self-identifying as American Indian/Alaskan Native women came exclusively from WHI, and were not living on American Indian/Alaskan Native reservations at the time of examination.

The Atherosclerosis Risk in Communities Study (ARIC)

ARIC is a prospective population-based study of four U.S. communities [9]. It was designed to investigate the causes of atherosclerosis and its clinical outcomes, as well as the key components (e.g. race, gender, geographic location, time period) of variation in CVD burden, and health care utilization. The larger ARIC study includes two separate parts: The Cohort Component and the Community Surveillance Component. The Cohort Component started in 1987 when the four ARIC field centers randomly selected, recruited approximately 4,000 individuals aged 45-64 years from each center, and began regular telephone follow-up of the cohort for health status updates. Weight, height, and several other descriptive factors were measured or self-reported as part of the ARIC cohort examinations. All ARIC cohort study participants provided written informed consent. Only the consenting African American subjects of the original ARIC cohort were genotyped on the MetaboChip.

In ARIC information on AAM was collected via questionnaire using the question: ‘Approximately how old were you when your menstrual periods started?’ ANM was determined via a question about the approximate timing of menopause (i.e. “At approximately what age did menopause begin?”) and if this onset was natural, or related to surgery, radiation, or unknown.

Epidemiologic Architecture for Genes Linked to Environment study accessing BioVU (EAGLE BioVU)

BioVU is Vanderbilt University Medical Center's biorepository of DNA extracted from discarded blood that was collected during routine clinical testing and then linked to de-identified health records available in the Synthetic Derivative, which contains highly detailed longitudinal clinical data for approximately two million patients, and is updated regularly to include new patients and append new data [10,11]. Planning for BioVU began in mid-2004 under the goal of providing a resource to investigators for studies of genotype-phenotype associations, and the first BioVU samples were collected in February 2007 at an accrual rate of ~500-700 samples per week. BioVU uses an "opt out" model, which was informed by an opinion from the federal Office of Human Research Protection (OHRP) that discarded biologic samples could be used and linked to de-identified clinical data for biomedical research without having to obtain prospective consenting of each individual [10,11]. The Epidemiologic Architecture for Genes Linked to Environment (EAGLE) study accessed all non-European descent patients as of 2011 [12]. The Vanderbilt University Center for Human Genetics Research (CHGR) DNA Resources Core genotyped these samples along with 360 HapMap samples on the MetaboChip [13]. Race/ethnicity was administratively assigned in BioVU assigned as previously described [2,3]. As described previously [14], an algorithm was used to identify the AAM and ANM in medical records from EAGLE BioVU, and extracted covariates correspond to the specific clinic visit pulled by this algorithm. Using this data mining approach, we only had sufficient sample size to include African American women with AAM in EAGLE BioVU (n>50).

The Coronary Artery Risk Development in Young Adults Study (CARDIA)

CARDIA began in 1985-1986 with a group of 5115 black and white men and women aged 18-30 years to examine the determinants of CVD and its risk factors [15]. The CARDIA

participants were selected to equally represent a number of subgroups of race, gender, education (high school or less and more than high school) and age (18-24 and 25-30) across four centers: Birmingham, AL; Chicago, IL; Minneapolis, MN; and Oakland, CA. Participants were invited to participate in follow-up examinations during 1987-1988 (Year 2), 1990-1991 (Year 5), 1992-1993 (Year 7), 1995-1996 (Year 10), 2000-2001 (Year 15), and 2005-2006 (Year 20), yielding retention rates from 72-90% across all follow-ups. The CARDIA examinations have collected medical and family histories, several CVD risk factors and anthropometrics. The participants in the CARDIA cohort were born between 1955-1968 and provide a unique avenue to investigate the mechanisms linking obesity to derangements in CVD in individuals earlier in the life course. All CARDIA study participants provided written informed consent. Only African American women from CARDIA were included in this analysis of MetaboChip data.

In CARDIA, AAM was collected via questionnaire using the question: 'How old were you when you began menstruating?' Then ANM was determined via several questions about factors surrounding a woman's experience of menopause (i.e. "Have you undergone menopause? Have you gone through menopause or the change of life? How old were you when this occurred? How did your periods stop?").

The Hispanic Community Health Study / Study of Latinos (HCHS/SOL)

The Hispanic Community Health Study/Study of Latinos (HCHS/SOL) is a population-based study of four urban Hispanic/Latino communities that was designed to identify CVD risk factors playing a protective or harmful role in Hispanics/Latinos, including acculturation [16]. The target population of HCHS/SOL included 16,000 adults (18-74 years at screening) of Hispanic/Latino origin, specifically of Cuban, Puerto Rican, Mexican, and Central/South American heritage, who were living at one of four field centers affiliated with San Diego State University, Northwestern University in Chicago, Albert Einstein College of Medicine in the Bronx area of New York, and the University of Miami. Seven additional academic centers serve as

scientific and logistical support centers, including the HCHS/SOL CC at the University of North Carolina at Chapel Hill. The HCHS/SOL participants underwent an extensive baseline clinic exam between 2008-2011, follow-up examination (ongoing), and annual follow-up interviews are ongoing to determine health outcomes of interest.

In HCHS/SOL, AAM and onset of menopause were assessed by questionnaire in English or Spanish based on the participant's preference (e.g. "At what age did your menses begin? Have you reached menopause (change of life)? At what age?") as part of the medical history of the baseline examination. If study personnel were asked to clarify what menopause or the change of life, then the definition included the requirement of cessation of periods for more than 12 months. Therefore, women reporting menopausal ages less than one year prior to their current age were excluded. Additionally, to exclude surgical onset of menopause we also excluded women who had a hysterectomy prior to or in the same year as their reported age at menopause, as well as women who reported having received a hysterectomy and having experienced menopause, but were missing information on the age at hysterectomy. Among the women reporting a hysterectomy, women also reported if they had a bilateral or unilateral oophorectomy. However, this step-wise approach may not have identified women who experienced oophorectomies in the absence of a hysterectomy. Data on the timing of radiologic or hormone replacement therapy prior to menopausal onset were not collected as part of the baseline examination.

Hypertension Genetic Epidemiology Network (HyperGEN)

HyperGEN is part of the Family Blood Pressure Program designed to study the genetic underpinnings of hypertension and other related conditions. Participants were recruited from multiply-affected hypertensive sib-ships, which were ascertained through population-based cohorts or from the community-at-large. The study was later extended to include normotensive offspring of the original hypertensive sibling pairs. Hypertensive sibships were defined as having

two or more siblings diagnosed with hypertension before age 60. Participants with type 1 diabetes or advanced renal disease (defined as serum creatinine level >2 mg/dL) were excluded. By 2003 two of four centers (AL, NC) recruited 1,264 African Americans, while three centers (NC, MN, and UT) recruited European Americans [17]. All study participants provided written informed consent, and all African American participants were genotyped on the MetaboChip for this analysis.

In HyperGEN ANM was measured via questionnaire ("Have you reached menopause? Age of menopause? Cause of menopause?") and only 'natural' causes of menopause were included in the current analysis.

The Multiethnic Cohort Study of Diet and Cancer (MEC)

The MEC was established in 1993 to examine lifestyle risk factors and genetic susceptibility for cancer and CVD in five racial/ethnic groups at the University of Hawai'i Cancer Center, in Honolulu, HI, and the Keck School of Medicine, University of Southern California (USC) in Los Angeles, CA [18,19]. The MEC cohort is comprised of more than 215,000 men and women primarily of African American, Japanese, Latino, Native Hawaiian and European ancestry. Every cohort member completed a self-administered 26-page baseline questionnaire at entry to the MEC Study (1993-1996), which included an extensive diet history, demographics, medical, medication, physical activity and female reproductive histories. Incident cancer cases are identified through cancer registries that have been established by state statute in Hawai'i and California. In addition to the baseline questionnaire, two additional questionnaires were mailed to MEC participants including a 4-page questionnaire that was sent in 1999-2001 and another 26-page questionnaire that was sent in 2003-2008. Biological specimens were collected from selected members of the cohort, starting in 1996, but more concertedly from 2001-2006. Subjects from the MEC cohort were selected in waves for MetaboChip based on their availability of biomarker for CVD risk factors or other clinical characteristics. Type 2 diabetes

cases and controls were genotyped as part of the Slim Initiative in Genomic Medicine for the Americas Type 2 Diabetes Consortium (SIGMA) and then imputed to the MetaboChip SNPs [4].

In MEC, both AAM and ANM were obtained by questionnaire and collected in a categorical manner. AAM was assessed by the question “How old were you when you had your first menstrual period?” and the following categories: 1=<11, 2=11-12, 3=13-14, 4=15-16, 5=17 or older. ANM was assessed by several questions “Have your menstrual periods stopped permanently? If yes, how old were you when this happened?” and the following categories: 1=<40, 2=40-44, 3=45-49, 4=50-54, 5=55 or older. Then we excluded all reported (“If yes, for what reason did your menstrual periods stop?”) non-natural causes of menopause (e.g. survey, radiation, medication).

The Multi-Ethnic Study of Atherosclerosis (MESA)

MESA is an ongoing study of subclinical cardiovascular disease and the risk factors that may predict progression to clinically-overt cardiovascular disease [20]. The MESA population-based cohort included a sample of 6,814 asymptomatic men and women aged 45-84. None had known heart disease at enrollment. Subjects were enrolled at Columbia University (New York City), Johns Hopkins University (Baltimore, MD), Northwestern University (Chicago, IL), University of Minnesota (Minneapolis-St. Paul), University of California at Los Angeles, and Wake Forest University (Winston-Salem, NC). Approximately 38 percent of the sample identified as white, 28 percent as African-American, 22 percent as Hispanic/Latino, and 12 percent as Asian (predominantly of Chinese descent). The first MESA examination took place over two years, from July 2000-July 2002, and the next three examinations were 17-20 months in length. The most recent examination occurred between April 2010 and January 2012. Participants are regularly contacted as part of an annual follow up to assess health outcomes.

In MESA, ANM was assessed via questionnaire by the following questions: Have you gone through menopause (change of life)? At what age did you go through menopause? Have you had a hysterectomy (surgery to remove your uterus/womb)? If Yes: at what age? Have you had surgery to remove your ovaries? If Yes: at what age? How many ovaries were removed? Have you ever taken hormone replacement therapy? Women reporting hysterectomy, oophorectomy or hormone replacement therapy prior to or equal to the reported age at menopause were excluded from analysis.

Mount Sinai Biobank Program (BioME)

The BioMe Biobank is an ongoing, prospective, hospital- and outpatient- based population research program operated by The Charles Bronfman Institute for Personalized Medicine (IPM) at Mount Sinai and has enrolled over 33,000 participants since September 2007 [6]. BioMe is an Electronic Medical Record (EMR)-linked biobank that integrates research data and clinical care information for consented patients at The Mount Sinai Medical Center, which serves diverse local communities of upper Manhattan with broad health disparities. BioMe populations include 25% of African Ancestry, 36% of Hispanic/Latino ancestry, 30% of European Ancestry, and 9% of other ancestry. The BioMe disease burden is reflective of health disparities in the local communities. BioMe operations are fully integrated in clinical care processes, including direct recruitment from clinical sites waiting areas and phlebotomy stations by dedicated recruiters independent of clinical care providers, prior to or following a clinician standard of care visit. Recruitment currently occurs at a broad spectrum of over 30 clinical care sites.

As described previously [14], the algorithm that was developed originally for EAGLE BioVU was also used to identify the AAM and ANM in the BioMe medical records. Similarly, covariates corresponded to the specific clinic visit pulled by this algorithm. Using this approach,

we had sufficient sample sizes to include African American (AAM only) and Hispanic/Latina women (AAM and ANM) in BioMe (N>50).

The Women's Health Initiative (WHI)

The WHI is a large study of postmenopausal women's health investigating risk factors for cancer, CVD, age-related fractures and chronic disease [21]. It began in 1993 as a set of randomized controlled clinical trials (CT) and an observational study (OS). Specifically, the CT (n=68,132) included three overlapping components: The Hormone Therapy (HT) Trials (n=27,347), Dietary Modification (DM) Trial (n=48,835), and Calcium and Vitamin D (CaD) Trial (n=36,282). Eligible women could be randomized into as many as all three CTs components. Women who were ineligible or unwilling to join the CT were then invited to join the OS (n=93,676). All WHI participants included in these analyses provided informed consent to submit their genotype data to dbGaP and were either directly genotyped on the MetaboChip or had previously-collected genome-wide data (Affymetrix 6.0 array) available for imputation (details, see Methods section in main text).

In WHI, AAM was assessed by questionnaire and collected in a categorical manner. AAM was assessed by the question "How old were you when you had your first menstrual period (period)?" and the following categories: 9 or less, 10, 11, 12, 13, 14, 15, 16, 17 or older. ANM was assessed by the question "How old were you when you last had regular menstrual bleeding (a period)?". Women were excluded if 1) the age at last bleeding was < 40 or > 60 years of age, 2) hormone therapy (for menopausal symptoms such as hot flashes or night sweats, following hysterectomy with removal of the ovaries or for the prevention of bone loss) was started before the reported age at last bleeding, 3) a bilateral oophorectomy occurred before the age at last bleeding (the age at oophorectomy was reported as a range, so if the age of last bleeding was within the range, the woman was excluded), 4) a hysterectomy occurred

before the age at last bleeding (the age of hysterectomy was also reported as a range, so if the age of hysterectomy was within the range, the woman was excluded).

REFERENCES

1. Matise TC, Ambite JL, Buyske S, Carlson CS, Cole SA, et al. (2011) The Next PAGE in understanding complex traits: design for the analysis of Population Architecture Using Genetics and Epidemiology (PAGE) Study. Am J Epidemiol 174: 849-859.
2. Dumitrescu L, Ritchie MD, Brown-Gentry K, Pulley JM, Basford M, et al. (2010) Assessing the accuracy of observer-reported ancestry in a biorepository linked to electronic medical records. Genet Med 12: 648-650.
3. Hall JB, Dumitrescu L, Dilks HH, Crawford DC, Bush WS (2014) Accuracy of administratively-assigned ancestry for diverse populations in an electronic medical record-linked biobank. PLoS One 9: e99161.
4. Consortium STD, Williams AL, Jacobs SB, Moreno-Macias H, Huerta-Chagoya A, et al. (2014) Sequence variants in SLC16A11 are a common risk factor for type 2 diabetes in Mexico. Nature 506: 97-101.
5. Daviglus ML, Talavera GA, Aviles-Santa ML, Allison M, Cai J, et al. (2012) Prevalence of major cardiovascular risk factors and cardiovascular diseases among Hispanic/Latino individuals of diverse backgrounds in the United States. JAMA 308: 1775-1784.
6. Tayo BO, Teil M, Tong L, Qin H, Khitrov G, et al. (2011) Genetic background of patients from a university medical center in Manhattan: implications for personalized medicine. PLoS One 6: e19166.
7. Conomos MP, Laurie CA, Stilp AM, Gogarten SM, McHugh CP, et al. (2016) Genetic Diversity and Association Studies in US Hispanic/Latino Populations: Applications in the Hispanic Community Health Study/Study of Latinos. Am J Hum Genet 98: 165-184.
8. Carty CL, Spencer KL, Setiawan VW, Fernandez-Rhodes L, Malinowski J, et al. (2013) Replication of genetic loci for ages at menarche and menopause in the multi-ethnic Population Architecture using Genomics and Epidemiology (PAGE) study. Human Reproduction 28: 1695-1706.
9. (1989) The Atherosclerosis Risk in Communities (ARIC) Study: design and objectives. The ARIC investigators. Am J Epidemiol 129: 687-702.
10. Pulley J, Clayton E, Bernard GR, Roden DM, Masys DR (2010) Principles of human subjects protections applied in an opt-out, de-identified biobank. Clin Transl Sci 3: 42-48.
11. Roden DM, Pulley JM, Basford MA, Bernard GR, Clayton EW, et al. (2008) Development of a large-scale de-identified DNA biobank to enable personalized medicine. Clin Pharmacol Ther 84: 362-369.
12. Crawford DC, Goodloe R, Farber-Eger E, Boston J, Pendergrass SA, et al. (2015) Leveraging Epidemiologic and Clinical Collections for Genomic Studies of Complex Traits. Hum Hered 79: 137-146.
13. Crawford DC, Goodloe R, Brown-Gentry K, Wilson S, Roberson J, et al. (2013) Characterization of the Metabochip in diverse populations from the International HapMap Project in the Epidemiologic Architecture for Genes Linked to Environment (EAGLE) project. Pac Symp Biocomput: 188-199.
14. Malinowski J, Farber-Eger E, Crawford DC (2014) Development of a data-mining algorithm to identify ages at reproductive milestones in electronic medical records. Pac Symp Biocomput: 376-387.
15. Friedman GD, Cutter GR, Donahue RP, Hughes GH, Hulley SB, et al. (1988) CARDIA: study design, recruitment, and some characteristics of the examined subjects. J Clin Epidemiol 41: 1105-1116.
16. Sorlie PD, Aviles-Santa LM, Wassertheil-Smoller S, Kaplan RC, Daviglus ML, et al. (2010) Design and implementation of the Hispanic Community Health Study/Study of Latinos. Ann Epidemiol 20: 629-641.

17. Williams RR, Rao DC, Ellison RC, Arnett DK, Heiss G, et al. (2000) NHLBI family blood pressure program: methodology and recruitment in the HyperGEN network. Hypertension genetic epidemiology network. Ann Epidemiol 10: 389-400.
18. Kolonel LN, Altshuler D, Henderson BE (2004) The multiethnic cohort study: exploring genes, lifestyle and cancer risk. Nat Rev Cancer 4: 519-527.
19. Lim U, Ernst T, Buchthal S, Latch M, Albright CL, et al. (2011) Asian Women Have Greater Abdominal and Visceral Adiposity Than Caucasian Women With Similar Body Mass Index. Obesity 19: S224-S224.
20. Bild DE, Bluemke DA, Burke GL, Detrano R, Diez Roux AV, et al. (2002) Multi-Ethnic Study of Atherosclerosis: objectives and design. Am J Epidemiol 156: 871-881.
21. Anderson G, Cummings S, Freedman LS, Furberg C, Henderson M, et al. (1998) Design of the Women's Health Initiative Clinical Trial and Observational Study. Controlled Clinical Trials 19: 61-109.
