# Supplementary material for: The genetic underpinnings of variation in ages at menarche and natural menopause among women from the multi-ethnic Population Architecture using Genomics and Epidemiology (PAGE) Study: A trans-ethnic meta-analysis
Source: PLoS One. 2018 Jul 25;13(7):e0200486. doi: 10.1371/journal.pone.0200486 (PMC6059436; doi:10.1371/journal.pone.0200486)
Supplement: S2 Text — (PDF) [file pone.0200486.s002.pdf]

S2 TEXT

SUPPLEMENTAL METHODS

Definition of Reproductive Phenotypes

Self-reported age at menarche (AAM, onset of first menses) and age at menopause (cessation of regular menses) were collected by questionnaire with the exception of two studies [the Vanderbilt University Medical Center's DNA biobank (EAGLE BioVU) and the Sinai Biobank Program (BioME)], which used an algorithm to obtain a woman's age of onset in years in the medical record [1]. Women reported their AAM or age at menopause in years. AAM and age at natural menopause (ANM) were harmonized across the studies as reported previously [2]. Briefly, two Population Architecture using Genomics and Epidemiology (PAGE) studies had used categories in their questionnaires to collect this information [AAM in the Multiethnic Cohort Study (MEC) and the Women's Health Initiative, (WHI), ANM in MEC]. As shown in the Figure below, a pseudo-continuous coding (based on the anticipated midpoint of a given interval) was used to harmonize across studies.

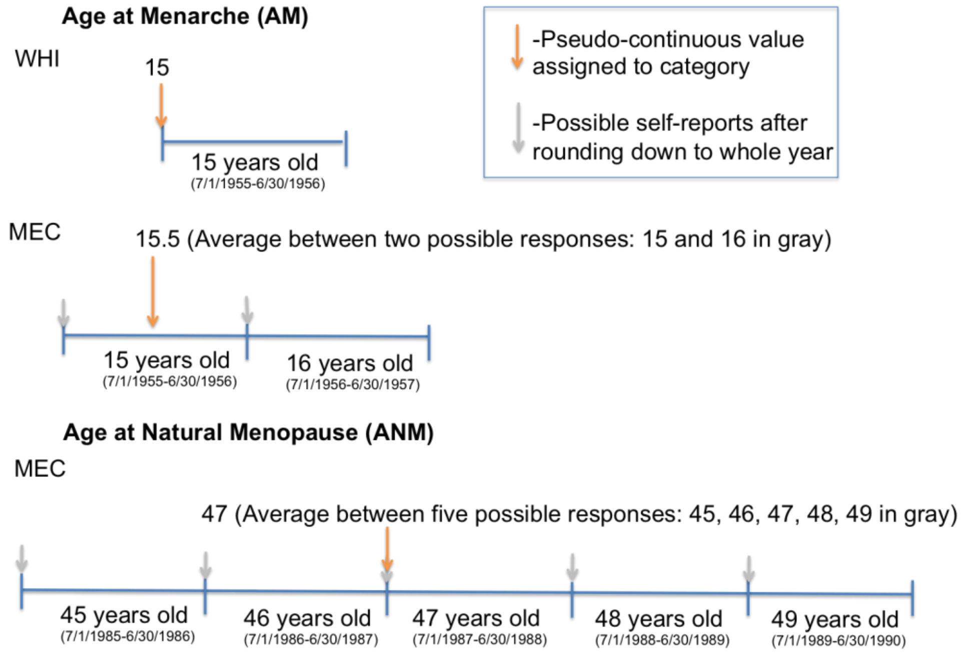

Figure. Coding schematic for pseudo continuous definition of ages at menarche and natural menopause

To minimize the effect of outliers and possible recording errors in AAM and ANM, women reporting an AAM beyond three standard deviations from the race/ethnic-study-specific mean AAM were excluded, and women were excluded who reported ANM values <40 years, or >60 years (except for the MEC, where the highest category collected was ≥55 years). Additional exclusions were applied to ANM to exclude non-natural causes of menopause onset, as previously described [2,3]. For example, several studies [the Atherosclerosis Risk in Communities Study (ARIC), CARDIA (Coronary Artery Risk Development in Young Adults Study), HyperGEN (Hypertension Genetic Epidemiology Network), MEC] differentiated on their questionnaires between natural and non-natural causes of menopause (e.g. from surgery, radiation, medication), while other studies applied exclusions for factors that occurred prior to natural onset: hysterectomy, bilateral oophorectomy, hormone replacement therapy, and/or chemotherapy. The Hispanic Community Health Study/Study of Latinos (HCHS/SOL) questionnaire protocol defined menopause as the permanent cessation of menses for at least one year; therefore, any women self-reporting a menopausal onset inconsistent with this definition were also excluded from HCHS/SOL (e.g. <1 year without menses). Any women not reporting menopause at the time of examination were not included in this analysis.

Genotyping and Imputation

MetaboChip (Illumina, Inc.; San Diego, CA) genotyping for the PAGE Study was performed at several research genomics laboratories including: the Human Genetics Center of the University of Texas-Houston, the Vanderbilt University Center for Human Genetics Research (CHGR) DNA Resources Core, University of Southern California Genomics Core, and the Translational Genomics Research Institute [4]. To facilitate PAGE Study genotyping quality control, we typed 90 HapMap YRI (Yoruba in Ibadan, Nigeria) samples and for some studies, we also typed 2-3% study-specific blinded replicates. Genotyping of studies not part of the first phase of PAGE was done at the following locations: The Human Genetics Center of the University of Texas-Houston (HyperGEN), University of Southern California Genomics Core

(Slim Initiative in Genomic Medicine for the Americas Type 2 Diabetes Consortium, MEC-SIGMA), University of California—Los Angeles Clinical and Translational Science Institute Genotyping Core Laboratory at Cedars-Sinai (the Multi-Ethnic Study of Atherosclerosis, MESA), and at the Genomics Core Facility at the Icahn School of Medicine at Mount Sinai (BioME)].

The custom Illumina, Inc. iSELECT array, MetaboChip, genotyped 196,483 autosomal SNPs including the high-density genotyping of 257 regions associated with cardiometabolic traits as of 2009 [5]. Forty-six index SNPs were included due to previous associations with either AAM or ANM (or if unavailable on the MetaboChip, a proxy SNP $r^2 \geq 0.8$ in 1000 Genomes CEU sample) and for representing distinct genetic loci ($r^2 < 0.2$ in ARIC African Americans or HCHS/SOL Hispanic/Latinos) (S3 Table). These SNPs included all 2 of the known AAM and 5 of the known ANM loci as of 2009, including the two strongest and most widely-generalizable AAM and ANM signals to date (*LIN28B* and *MCM8*) [6,7].

Herein, we adopted the locus definitions used in the design of the MetaboChip [5]. The number of SNPs per densely genotyped reproductive trait locus therefore varied as a function of both the MetaboChip design tiered-prioritization of 11 cardiometabolic phenotypes of highest interest such as body mass index (BMI), triglycerides, or coronary artery disease [5], as well as the base pair range of the putative regions of interest (before quality control, 672 to 2,702 SNPs located within a locus range of 313kb to 1.2Mb, respectively; S4 Table). Thus, the MetaboChip includes six densely genotyped loci that were initially placed on the chip for their associations with BMI, but have since also been associated with AAM at different, or in some cases at the same SNP [8,9]. Additionally, a densely genotyped triglycerides locus on the MetaboChip has since been associated with ANM [10] and BMI [11].

In three studies, imputation of MetaboChip SNPs was conducted using 1000 Genomes reference samples [MEC SIGMA, BioME, WHI-SNP Health Association Resource African Americans [12]]. SNP imputations were filtered by imputation quality score (MEC SIGMA based

on info from IMPUTE2; BioME based on proper_info from SNPTEST; WHI based on the Rsq from MaCH); only high-quality SNPs were included in this analysis.

PLINK was used to identify related participants across studies of the first wave of PAGE [13]. Individuals with high inbreeding coefficients, $F > 0.15$, were excluded [14]. The PAGE Study calculated principal components using Eigensoft [15,16] in an unrelated subset of each study, or using 1000 Genomes reference populations (CEU, YRI, MXL, PUR, CLM and CHB) in HCHS/SOL [17], to then project to the study's entire sample. Then using the resulting principal components, we excluded individuals that were ancestral outliers for their study- and race/ethnic-specific group [4].

Person-Level Quality Control

Within each of the PAGE sub-studies (ARIC, EAGLE BioVU, CARDIA, HCHS/SOL, MEC, WHI) duplicate samples, ancestry outliers, samples with phenotype-genotype sex discordance, low person-level call rate (<95%), excessive heterozygosity, or the sample in a 1st degree relative pair with the lowest call rate were excluded from all analyses with one exception. If a woman from WHI was in a 1st degree relative pair with another woman from HCHS/SOL, then the post-menopausal WHI participant was preferentially retained. Collaborating studies not part of the first phase of the PAGE Study (HyperGen, MEC SIGMA, MESA, BioME) applied similar person-level filters within each of their studies. Only study- and race/ethnic-specific sample sizes of 50 women or more were carried forward to statistical analyses and summarized descriptively in S1-2 Tables.

Collectively, the studies represent 20,398 African American, 15,856 Hispanic/Latina, 8,572 Asian American, and 538 American Indian/Alaskan Native women who provided informed consent (or in the case of EAGLE BioVU, did not opt out from the approved research), and had complete information on genetics, reproductive phenotypes and covariates (AAM: S1 Table; ANM: S2 Table).

SNP-Level Quality Control

Within each racial/ethnic group, SNPs on the X chromosome or those with poor genotype quality (<95% call rate, <0.4 cluster separation scores, <0.6 GenTrain score, or with otherwise poor quality) were excluded. Exclusions for low study and race/ethnic-specific minor allele frequency (MAF) <0.1% and deviations from Hardy-Weinberg Equilibrium, p-value<$1x10^{-6}$, were applied within each race/ethnic and study group with one modification. SNPs in Hispanic/Latino samples, including the MEC SIGMA Type 2 Diabetes case-control study, were excluded only if they failed Hardy-Weinberg Equilibrium in the entire HCHS/SOL sample, as well as in at least one Hispanic/Latino background group captured in the HCHS/SOL (747 SNPs excluded).

Statistical Modeling and Analyses

The association between a SNP and AAM or ANM was modeled for each study under an additive genetic model and adjusted for birth year (or approximation thereof, in the case of HCHS/SOL and WHI), the top 10 principal components for ancestry, and center/region (if applicable). MEC and WHI samples were further stratified by PAGE genotyping wave or imputation. Additionally, given the study design, MEC SIGMA was stratified by Type 2 diabetes case/control status.

In HCHS/SOL generalized estimating equations were implemented using SUGEN 6.2 to account for clustering and relatedness within households as well as the complex sampling design [17]. The Genome-Wide Association analyses with Family Data package was used in R (https://www.r-project.org/) to account for relatedness in HyperGEN using a linear mixed model [18]. In all other studies, linear regression models were implemented using PLINK v1.07 [13] or R (https://cran.r-project.org).

Within each racial/ethnic group we implemented fixed-effect inverse-variance weighted meta-analyses using METAL (version from 2011-03-25) [19]. After applying the above SNP-level quality controls filters, and requiring that the race/ethnic-specific estimates were informed by at least half of the maximum sample size, between 123,493 and 157,710 MetaboChip SNP fixed-effect estimates remained across the racial/ethnic groups.

Data Visualization

Regional plots were generated in LocusZoom [20] to visualize the trans-ethnic modified random-effects SNP associations and linkage disequilibrium patterns, based on two trans-ethnic samples of 13,405 and 14,448 individuals from HCHS/SOL (men and women) and WHI that corresponded to the proportions of African, Hispanic/Latina, and Asian descent individuals included in the final trans-ethnic AAM and ANM meta-analyses, respectively.

Post Hoc Power Calculations

To aid with the interpretation of study findings, we conducted *post hoc* statistical power calculations in Quanto version 1.2.4 [21] for AAM and ANM genetic effects. Using information available on the varying sample sizes of the race/ethnic specific and trans-ethnic meta-analyses results, the mean and standard deviation observed in the largest study sample with either quantitative AAM and ANM (S1-2 Tables), and the Bonferroni corrections for the number of loci investigated per trait using index SNPs or their proxies, we calculated power to detect effects up to a 0.13-year decrease in AAM or a up to a 0.20-year decrease in ANM per allele, which corresponds to the most recent validated effects of *LIN28B* and for *MCM8* on AAM and ANM among European descent women [6,7]. Note that SNPs within densely genotyped regions that are not the index SNP or its proxy (S3-4 Tables), are held to a locus-specific threshold that is intermediate to the more stringent array-wide and the less stringent Bonferroni threshold for index/proxy SNPs, and are not presented in the power calculations below.

Power to detect the effect magnitude of *LIN28B* in the trans-ethnic meta-analysis of AAM allowed for the identification of genetic effects at ≥80% power for common variants ≥9% allele frequency at array-wide and ≥4% allele frequency at Bonferroni significance for the number of previously reported variants tested. The trans-ethnic meta-analysis of ANM for the smallest effect size recently reported for *MCM8* [6], was less powered, never reaching ≥80% power for array-wide significance and reaching ≥80% power Bonferroni significance for MAF≥30%. Across racial/ethnic groups, the power to detect these true positive effects varied with the varying sample size of each group in the AAM and ANM analyses. For example, with AAM the African, Hispanic/Latino and Asian American meta-analyses could describe effects of the size of *LIN28B* with ≥80% power at a MAF≥9%, 12% and 26%, respectively.

REFERENCES

1. Malinowski J, Farber-Eger E, Crawford DC (2014) Development of a data-mining algorithm to identify ages at reproductive milestones in electronic medical records. Pac Symp Biocomput: 376-387.
2. Carty CL, Spencer KL, Setiawan VW, Fernandez-Rhodes L, Malinowski J, et al. (2013) Replication of genetic loci for ages at menarche and menopause in the multi-ethnic Population Architecture using Genomics and Epidemiology (PAGE) study. Human Reproduction 28: 1695-1706.
3. Spencer KL, Malinowski J, Carty CL, Franceschini N, Fernandez-Rhodes L, et al. (2013) Genetic variation and reproductive timing: African American women from the Population Architecture using Genomics and Epidemiology (PAGE) Study. PLoS One 8: e55258.
4. Buyske S, Wu Y, Carty CL, Cheng I, Assimes TL, et al. (2012) Evaluation of the metabochip genotyping array in African Americans and implications for fine mapping of GWAS-identified loci: the PAGE study. PLoS One 7: e35651.
5. Voight BF, Kang HM, Ding J, Palmer CD, Sidore C, et al. (2012) The metabochip, a custom genotyping array for genetic studies of metabolic, cardiovascular, and anthropometric traits. PLoS Genet 8: e1002793.
6. Day FR, Ruth KS, Thompson DJ, Lunetta KL, Pervjakova N, et al. (2015) Large-scale genomic analyses link reproductive aging to hypothalamic signaling, breast cancer susceptibility and BRCA1-mediated DNA repair. Nat Genet 47: 1294-1303.
7. Day FR, Thompson DJ, Helgason H, Chasman DI, Finucane H, et al. (2017) Genomic analyses identify hundreds of variants associated with age at menarche and support a role for puberty timing in cancer risk. Nat Genet.
8. Elks CE, Perry JR, Sulem P, Chasman DI, Franceschini N, et al. (2010) Thirty new loci for age at menarche identified by a meta-analysis of genome-wide association studies. Nat Genet 42: 1077-1085.
9. Perry JR, Day F, Elks CE, Sulem P, Thompson DJ, et al. (2014) Parent-of-origin-specific allelic associations among 106 genomic loci for age at menarche. Nature 514: 92-97.
10. Stolk L, Perry JR, Chasman DI, He C, Mangino M, et al. (2012) Meta-analyses identify 13 loci associated with age at menopause and highlight DNA repair and immune pathways. Nat Genet 44: 260-268.
11. Gong J, Nishimura KK, Fernandez-Rhodes L, Haessler J, Bien S, et al. (2018) Trans-ethnic analysis of metabochip data identifies two new loci associated with BMI. Int J Obes (Lond) 42: 384-390.
12. Liu EY, Buyske S, Aragaki AK, Peters U, Boerwinkle E, et al. (2012) Genotype imputation of Metabochip SNPs using a study-specific reference panel of ~4,000 haplotypes in African Americans from the Women's Health Initiative. Genet Epidemiol 36: 107-117.
13. Purcell S, Neale B, Todd-Brown K, Thomas L, Ferreira MA, et al. (2007) PLINK: a tool set for whole-genome association and population-based linkage analyses. Am J Hum Genet 81: 559-575.
14. Weale ME (2010) Quality control for genome-wide association studies. Methods Mol Biol 628: 341-372.
15. Patterson N, Price AL, Reich D (2006) Population structure and eigenanalysis. PLoS Genet 2: e190.
16. Price AL, Patterson NJ, Plenge RM, Weinblatt ME, Shadick NA, et al. (2006) Principal components analysis corrects for stratification in genome-wide association studies. Nat Genet 38: 904-909.
17. Lin DY, Tao R, Kalsbeek WD, Zeng DL, Gonzalez F, et al. (2014) Genetic Association Analysis under Complex Survey Sampling: The Hispanic Community Health Study/Study of Latinos. Am J Hum Genet 95: 675-688.

18. Chen MH, Yang Q (2010) GWAF: an R package for genome-wide association analyses with family data. Bioinformatics 26: 580-581.
19. Willer CJ, Li Y, Abecasis GR (2010) METAL: fast and efficient meta-analysis of genomewide association scans. Bioinformatics 26: 2190-2191.
20. Pruim RJ, Welch RP, Sanna S, Teslovich TM, Chines PS, et al. (2010) LocusZoom: regional visualization of genome-wide association scan results. Bioinformatics 26: 2336-2337.
21. Gauderman WJ, Morrison JM (2006) QUANTO 1.1: A computer program for power and sample size calculations for genetic-epidemiology studies.
