# Supplementary material for: The genetic underpinnings of variation in ages at menarche and natural menopause among women from the multi-ethnic Population Architecture using Genomics and Epidemiology (PAGE) Study: A trans-ethnic meta-analysis
Source: PLoS One. 2018 Jul 25;13(7):e0200486. doi: 10.1371/journal.pone.0200486 (PMC6059436; doi:10.1371/journal.pone.0200486)
Supplement: S1 Table — (PDF) [file pone.0200486.s003.pdf]

Supplemental Table 1: Evidence of generalization at 46 previously described* age at menarche and natural menopause signals across multiple race/ethnic groups

									African American							Hispanic/Latina American							Asian American							American Indian/Alaskan Native						Modified Random-Effects Trans-Ethnic***							
**GWAS/Array-Wide Significant Ref***	**SNP**	**GWAS Decreasing Allele****	**GWAS Increasing Allele****	**Unavailable Index SNP-Decreasing Allele**	**Chr**	**BP**	**Gene***	**Coded Allele**	**Freq**	**Beta (years)**	**SE**	**P-value**	**P-value heterogeneity**	**N**	**Directional Consistency**	**Freq**	**Beta (years)**	**SE**	**P-value**	**P-value heterogeneity**	**N**	**Directional Consistency**	**Freq**	**Beta (years)**	**SE**	**P-value**	**P-value heterogeneity**	**N**	**Directional Consistency**	**Freq**	**Beta (years)**	**SE**	**P-value**	**N**	**Directional Consistency**	**Freq**	**Freq Range**	**Beta (years)**	**SE**	**Modified P-value**	**N**	**N Studies**	**Direct-ional Consis-tency**
**MENARCHE**																																											
Perry *et al*., 2014 (Nature)	rs660899**	T	G	rs2274465-G	1	44117006	*KDM4A, PTPRF*	T	0.210	-0.018	0.02	0.378	0.995	20146	1	0.247	-0.055	0.02	*0.020*	0.100	15347	1	0.253	-0.027	0.03	0.339	0.512	8273	1	0.263	-0.193	0.13	0.134	538	1	0.227	0.17 - 0.31	-0.032	0.01	*0.030*	43766	22	1
Perry *et al*., 2014 (Nature)	rs10889947**	C	T	rs3101336-C	1	72828221	*NEGR1*	C	0.337	0.016	0.02	0.367	0.583	20200	0	0.680	-0.004	0.02	0.858	0.865	15336	1	0.893	-0.009	0.04	0.827	0.980	8253	1	0.608	0.040	0.11	0.716	538	0	0.569	0.32 - 0.91	0.006	0.01	0.726	43789	22	0
Perry *et al*., 2014 (Nature)	rs3845344**	T	C	rs7514705-T	1	75001480	*TNNI3K, TYW3*	T	0.135	0.054	0.03	*0.032*	0.288	20209	0	0.461	-0.016	0.02	0.448	0.346	15345	1	0.692	-0.015	0.03	0.562	0.817	8272	1	0.404	-0.119	0.12	0.303	538	1	0.362	0.11 - 0.73	0.009	0.02	0.361	43826	22	0
Elks *et al*., 2010	rs633715	C	T	-	1	177852580	*SEC16B*	C	0.102	-0.013	0.03	0.668	0.142	15987	1	0.183	-0.066	0.03	*0.013*	0.586	15346	1	0.204	-0.013	0.03	0.684	0.164	8272	1	0.164	0.012	0.15	0.938	538	0	0.149	0.09 - 0.23	-0.035	0.02	0.064	39605	19	1
Perry *et al*., 2014 (Nature)	rs823114**	G	A	rs951366-C	1	205719532	***NUCKS1, RAB7L1***	G	0.754	-0.044	0.02	*0.024*	0.146	20199	1	0.589	-0.064	0.02	*0.002*	0.098	15343	1	0.469	-0.008	0.02	0.744	0.819	8266	1	0.568	-0.100	0.11	0.382	538	1	0.636	0.38 - 0.80	-0.042	0.02	**1.36E-03**	43808	22	1
Elks *et al*., 2010	rs2947411	G	A	-	2	614168	*TMEM18*	G	0.773	0.033	0.02	0.095	0.635	20205	0	0.846	-0.033	0.03	0.240	0.669	15346	1	0.907	-0.132	0.04	*0.002*	0.731	8259	1	0.816	0.010	0.15	0.946	538	0	0.831	0.75 - 0.94	-0.008	0.02	0.189	43810	22	1
Perry *et al*., 2014 (Nature)	rs6551278**	C	T	rs9849248-T	3	88199298	*ZNF654, HTR1F*	C	0.622	-0.002	0.02	0.916	0.895	20203	1	0.832	-0.042	0.03	0.125	0.243	15346	1	0.893	-0.030	0.04	0.463	0.217	8271	1	0.818	-0.254	0.14	0.078	538	1	0.748	0.59 - 0.92	-0.015	0.01	0.362	43820	22	1
Elks *et al*., 2010	rs2600959**	G	A	rs6439371-A	3	132615464	*TMEM108, NPHP3*	G	0.367	0.012	0.02	0.482	0.302	20124	0	0.570	-0.009	0.02	0.651	0.574	15338	1	0.736	-0.019	0.03	0.488	0.855	8163	1	0.595	-0.043	0.11	0.693	537	1	0.516	0.34 - 0.79	-0.001	0.01	0.934	43625	22	1
Perry *et al*., 2014 (Nature)	rs17451107**	C	T	rs900400-C	3	156797609	*LEKR1, CCNL1*	C	0.344	-0.034	0.02	0.056	0.517	20207	1	0.415	-0.045	0.02	*0.027*	0.641	15345	1	0.462	-0.005	0.02	0.831	0.898	8268	1	0.379	-0.113	0.11	0.320	538	1	0.388	0.31 - 0.48	-0.031	0.01	*0.015*	43820	22	1
Perry *et al*., 2014 (Nature)	rs13130484**	T	C	rs10938397-G	4	45175691	*GNPDA2*	T	0.248	-0.046	0.02	*0.019*	0.764	20208	1	0.376	-0.029	0.02	0.165	0.518	15346	1	0.281	-0.008	0.03	0.765	0.319	8271	1	0.367	-0.013	0.11	0.909	538	1	0.294	0.22 - 0.40	-0.031	0.01	*0.022*	43825	22	1
Perry *et al*., 2014 (Nature)	rs2306802**	A	G	rs13135934-G	4	95186055	*SMARCAD1*	A	0.866	-0.040	0.03	0.117	0.850	20201	1	0.657	-0.005	0.02	0.810	0.751	15342	1	0.521	-0.003	0.02	0.894	0.946	8273	1	0.658	-0.072	0.11	0.529	538	1	0.718	0.46 - 0.89	-0.014	0.01	0.386	43816	22	1
Perry *et al*., 2014 (Nature)	rs1490212**	A	C	rs9447700-T	6	77180707	*IMPG1*	A	0.750	-0.017	0.02	0.403	0.708	20207	1	0.354	0.026	0.02	0.236	0.510	15347	0	0.410	-0.059	0.02	*0.017*	0.175	8270	1	0.336	-0.052	0.12	0.677	538	1	0.547	0.27 - 0.78	-0.015	0.01	0.389	43824	22	1
Perry *et al*., 2014 (Nature)	rs9373571**	T	A	rs239198-C	6	101133834	*SIM1, ASCC3*	T	0.854	0.040	0.03	0.156	0.870	15199	0	0.589	-0.033	0.02	0.113	0.889	15306	1								0.595	0.086	0.11	0.437	538	0	0.723	0.54 - 0.88	-0.007	0.02	0.747	30505	14	1
Perry *et al*., 2009 (Nat Gen); Elks *et al*., 2010	rs7759938	T	C	-	6	105378954	***LIN28B***	T	0.464	-0.080	0.02	**2.15E-06**	0.501	20206	1	0.719	-0.106	0.02	**3.22E-06**	0.671	15345	1	0.702	-0.171	0.03	**1.00E-10**	0.733	8269	1	0.699	-0.302	0.12	*0.011*	538	1	0.601	0.42 - 0.76	-0.106	0.01	**4.71E-18**	43820	22	1
Elks *et al*., 2010	rs9401888**	G	A	rs1361108-T	6	126877523	*C6orf173, TRMT11*	G	0.224	-0.051	0.02	*0.013*	0.229	20206	1	0.627	-0.039	0.02	0.071	0.742	15344	1	0.775	-0.006	0.07	0.927	*0.048*	5089	1	0.535	-0.267	0.12	0.029	538	1	0.484	0.19 - 0.99	-0.044	0.02	*0.005*	40639	21	1
Elks *et al*., 2010	rs6473015**	C	A	rs7821178-A	8	78178485	*PXMP3*	C	0.139	-0.020	0.02	0.427	0.637	20187	1	0.249	-0.013	0.02	0.580	0.908	15345	1	0.211	-0.026	0.03	0.388	0.637	8270	1	0.240	-0.058	0.12	0.639	538	1	0.192	0.13 - 0.28	-0.018	0.01	0.294	43802	22	1
Perry *et al*., 2014 (Nature)	rs2475357**	T	G	rs7865468-G	9	10270339	*PTPRD*	T	0.642	-0.027	0.02	0.127	0.631	20162	1	0.489	-0.040	0.02	0.049	0.461	15342	1	0.509	-0.020	0.02	0.416	0.862	8265	1	0.431	0.105	0.11	0.346	538	0	0.571	0.48 - 0.69	-0.030	0.01	*0.020*	43769	22	1
Perry *et al*., 2009 (Nat Gen); Elks *et al*., 2010	rs2090409	A	C	-	9	108967088	***TMEM38B***	A								0.325	-0.087	0.02	**7.72E-05**	0.728	15342	1	0.432	-0.034	0.02	0.165	0.061	8249	1	0.324	-0.043	0.11	0.700	538	1	0.358	0.30 - 0.47	-0.060	0.02	**1.35E-04**	28222	15	1
Perry *et al*., 2014 (Nature)	rs7870597**	C	T	rs11792861-C	9	111871864	*TMEM245*	C	0.069	-0.035	0.03	0.308	*0.008*	20196	1	0.275	-0.010	0.02	0.674	0.804	15342	1	0.268	-0.002	0.03	0.938	0.817	8264	1	0.275	0.009	0.12	0.942	538	0	0.175	0.04 - 0.30	-0.015	0.02	0.535	43802	22	1
Perry *et al*., 2014 (Nature)	rs7086481**	T	A	rs1915146-A	10	126844523	*CTBP2*	T	0.419	-0.027	0.02	0.108	0.781	20199	1	0.454	-0.054	0.02	0.010	0.223	15347	1	0.763	0.020	0.03	0.488	0.557	8269	0	0.504	-0.098	0.11	0.378	538	1	0.500	0.40 - 0.81	-0.028	0.01	*0.033*	43815	22	1
Elks *et al*., 2010	rs4929923	C	T	-	11	8639200	*TRIM66*	C	0.592	-0.017	0.02	0.354	0.908	20198	1	0.575	-0.021	0.02	0.310	0.576	15347	1	0.393	-0.056	0.02	*0.024*	0.393	8273	1	0.609	-0.153	0.11	0.174	538	1	0.561	0.37 - 0.90	-0.028	0.01	*0.037*	43818	22	1
Elks *et al*., 2010	rs900145	T	C	-	11	13293905	*ARNTL*	T	0.468	-0.005	0.02	0.788	0.423	20182	1	0.645	-0.015	0.02	0.486	0.400	15312	1	0.530	0.012	0.02	0.628	0.691	8258	0	0.658	0.009	0.12	0.941	537	0	0.555	0.44 - 0.76	-0.004	0.01	0.816	43752	22	1
Perry *et al*., 2014 (Nature)	rs7103411	T	C	-	11	27700125	*BDNF*	T	0.912	0.005	0.03	0.860	*0.010*	20207	0	0.818	-0.068	0.03	*0.009*	0.688	15346	1	0.604	-0.060	0.03	*0.018*	0.904	8272	1	0.836	-0.087	0.15	0.554	538	1	0.818	0.58 - 0.92	-0.045	0.02	*0.006*	43825	22	1
Elks *et al*., 2010	rs2450138**	C	T	rs10899489-C	11	77924870	*GAB2*	C	0.842	-0.062	0.02	*0.007*	0.738	20205	1	0.723	-0.015	0.02	0.514	0.935	15339	1	0.600	-0.029	0.03	0.255	0.089	8272	1	0.754	0.039	0.14	0.781	538	0	0.762	0.55 - 0.88	-0.036	0.01	*0.016*	43816	22	1
Perry *et al*., 2014 (Nature)	rs1254319**	G	A	rs1254337-A	14	60903757	*SIX6*	G	0.513	-0.021	0.02	0.204	0.353	20203	1	0.705	-0.033	0.02	0.148	0.928	15342	1	0.348	-0.027	0.03	0.303	0.379	8271	1	0.714	-0.085	0.13	0.499	538	1	0.539	0.32 - 0.74	-0.026	0.01	0.052	43816	22	1
Perry *et al*., 2014 (Nature)	rs1212911**	T	C	rs10144321-G	14	100917372	*DLK1, WDR25*	T	0.255	-0.016	0.02	0.413	0.204	20175	1	0.456	-0.053	0.02	*0.010*	0.650	15345	1	0.512	-0.018	0.02	0.466	0.114	8273	1	0.373	0.053	0.12	0.669	538	0	0.381	0.24 - 0.55	-0.028	0.01	*0.028*	43793	22	1
Elks *et al*., 2010	rs1659127	A	G	-	16	14388305	*MKL2*	A	0.304	0.021	0.02	0.263	0.530	20205	0	0.292	0.034	0.02	0.135	0.336	15345	0	0.490	0.042	0.02	0.080	0.433	8271	0	0.281	0.132	0.13	0.297	538	0	0.342	0.26 - 0.53	0.030	0.01	*0.024*	43821	22	0
Perry *et al*., 2014 (Nature)	rs12446632	G	A	-	16	19935389	*GPRC5B*	G	0.911	0.009	0.03	0.795	0.127	15212	0	0.915	-0.041	0.04	0.267	0.596	15319	1								0.891	-0.381	0.17	*0.025*	538	1	0.912	0.86 - 0.93	-0.017	0.03	0.665	30531	14	1
Elks *et al*., 2010	rs9939609	A	T	-	16	53820527	*FTO*	A	0.472	-0.006	0.02	0.773	0.341	14850	1	0.309	-0.038	0.02	0.08	0.084	15344	1	0.203	-0.080	0.03	*0.008*	0.601	8273	1	0.349	0.014	0.12	0.907	538	0	0.348	0.18 - 0.49	-0.029	0.02	*0.031*	38467	20	1
Elks *et al*., 2010	rs889398**	C	T	rs1364063-T	16	69556715	*NFAT5*	C	0.716	0.022	0.02	0.231	0.787	20193	0	0.659	-0.018	0.02	0.411	0.232	15347	1	0.783	-0.072	0.03	*0.027*	0.389	8194	1	0.690	0.001	0.12	0.992	538	0	0.711	0.58 - 0.89	-0.006	0.01	0.689	43734	22	1
Perry *et al*., 2014 (Nature)	rs244334**	A	G	rs244293-A	17	53204055	*STXBP4*	A	0.213	-0.036	0.02	0.078	0.852	20205	1	0.454	-0.001	0.02	0.979	0.766	15344	1	0.504	-0.030	0.02	0.220	0.360	8273	1	0.405	-0.161	0.12	0.163	538	1	0.350	0.19 - 0.54	-0.021	0.01	0.134	43822	22	1
Perry *et al*., 2014 (Nature)	rs2836961**	C	A	rs2836950-G	21	40627020	*BRWD1*	C	0.257	-0.023	0.02	0.248	0.812	19310	1	0.437	0.001	0.02	0.957	0.762	14640	0	0.239	-0.059	0.03	*0.047*	0.104	7770	1	0.405	0.044	0.12	0.702	535	0	0.311	0.19 - 0.47	-0.021	0.01	0.160	41720	18	1
**NATURAL MENOPAUSE**																																											
Stolk *et al*., 2012	rs1776133**	G	A	rs1635501-C	1	242025098	*EXO1*	G	0.307	-0.028	0.03	0.297	0.071	7288	1	0.323	-0.107	0.06	0.070	0.855	5271	1	0.225	-0.033	0.03	0.251	0.054	4347	1	0.349	-0.056	0.52	0.913	185	1	0.284	0.21 - 0.35	-0.038	0.03	0.067	16906	23	1
Stolk *et al*., 2012	rs2303369	T	C	-	2	27715416	*FNDC4*	T	0.369	-0.023	0.03	0.397	0.413	5362	1	0.500	-0.044	0.05	0.415	*0.015*	5269	1	0.206	-0.060	0.03	0.065	0.115	4348	1	0.500	-0.321	0.52	0.539	185	1	0.367	0.11 - 0.53	-0.038	0.03	0.058	14979	21	1
Stolk *et al*., 2012	rs4668368**	A	T	rs10183486-T	2	171926162	*TLK1*	A	0.369	-0.029	0.03	0.281	0.451	7294	1	0.697	0.056	0.06	0.354	0.157	5270	0	0.898	-0.060	0.04	0.157	0.239	4348	1	0.703	0.746	0.54	0.168	185	0	0.611	0.31 - 0.94	-0.019	0.03	0.300	16912	23	1
Stolk *et al*., 2012	rs17355209**	A	C	rs4693089-A	4	84453308	*HELQ*	A	0.444	-0.040	0.03	0.113	0.809	6704	1	0.428	-0.116	0.06	*0.044*	0.854	4737	1	0.340	-0.032	0.02	0.200	0.349	4046	1	0.430	0.409	0.47	0.386	185	0	0.400	0.30 - 0.48	-0.043	0.02	*0.020*	15487	20	1
Spencer *et al*., 2013***	rs181686584	C	A	-	5	74725808	*COL4A3BP*	C	0.997	0.431	1.13	0.703	0.817	4209	0	0.996	-0.693	0.5	0.165	0.836	4669	1														0.994	0.98 - <1.0	-0.546	0.36	0.184	9373	10	1
He et al., 2009; Stolk *et al*., 2012	rs149307**	C	T	rs365132-G	5	176471591	*UIMC1*	C	0.211	-0.037	0.03	0.239	0.893	7109	1	0.578	-0.022	0.05	0.692	0.081	5271	1	0.507	-0.052	0.02	*0.025*	0.161	4345	1	0.527	1.077	0.47	*0.024*	185	0	0.422	0.20 - 0.60	-0.044	0.02	*0.022*	16725	22	1
He et al., 2009; Stolk *et al*., 2012	rs2153157	G	A	-	6	10897488	*SYCP2L*	G	0.300	-0.005	0.03	0.855	0.501	7104	1	0.572	-0.031	0.06	0.575	0.641	5271	1	0.341	-0.047	0.03	0.058	0.357	4348	1	0.562	-0.199	0.51	0.699	185	1	0.412	0.28 - 0.61	-0.029	0.02	0.146	16723	22	1
Spencer *et al*., 2013***	rs79972789	C	G	-	11	2824573	*KCNQ1*	C	0.997	0.586	0.23	*0.010*	0.703	5026	0	0.978	-0.21	0.19	0.267	0.783	5271	1								0.984	-1.890	1.97	0.339	185	1	0.989	0.97 - <1.0	0.114	0.13	0.482	12452	15	0
Stolk *et al*., 2012	rs11841782**	A	G	rs4886238-G	13	61135632	*TDRD3*	A	0.726	0.012	0.03	0.676	0.206	7298	0	0.778	-0.077	0.07	0.251	0.105	5271	1	0.909	0.008	0.04	0.839	0.705	4347	0	0.722	-0.750	0.54	0.164	185	1	0.789	0.70 - 0.97	-0.023	0.03	0.974	16916	23	1
Stolk *et al*., 2009	rs12866444**	C	T	rs7333181-G	13	112239743	*LOC121793, ARHGEF7*	C	0.968	-0.021	0.07	0.770	0.797	7295	1	0.917	0.094	0.1	0.349	0.382	5271	0	0.941	0.010	0.06	0.859	0.602	4347	0	0.908	-1.035	0.89	0.245	185	1	0.950	0.03 - 0.10	0.014	0.04	0.810	16913	23	0
Stolk *et al*., 2012	rs12593363**	G	A	rs2307449-G	15	89785116	*POLG*	G	0.267	0.005	0.03	0.860	0.734	7289	0	0.400	0.024	0.06	0.669	*0.045*	5271	0	0.306	-0.026	0.03	0.310	*0.045*	4347	1	0.341	-0.751	0.49	0.125	185	1	0.311	0.24 - 0.42	-0.005	0.03	0.737	16907	23	1
Spencer *et al*., 2013***	rs189596789	G	A	-	19	11189620	*LDLR*	G	0.993	0.085	0.15	0.576	0.346	6715	0																												
He et al., 2009; Stolk *et al*., 2012	rs11668309**	T	C	rs1172822-T; rs11668344-A	19	55833460	***BRSK1, TMEM150B***	T	0.238	-0.031	0.03	0.278	0.258	7294	1	0.343	-0.207	0.06	**2.52E-04**	0.892	5271	1	0.145	-0.038	0.04	0.361	0.821	4348	1	0.376	0.175	0.50	0.729	185	0	0.241	0.04 - 0.36	-0.065	0.02	*0.011*	16913	23	1
Stolk *et al*., 2009	rs236114	C	A	-	20	5935385	***MCM8***	C	0.904	0.017	0.04	0.694	0.951	7108	0	0.832	-0.215	0.07	**2.99E-03**	0.408	5270	1	0.945	-0.100	0.10	0.303	0.722	4049	1	0.870	-0.278	0.75	0.710	185	1	0.901	0.82 - <1.0	-0.051	0.03	0.205	16427	21	1

*Genome-wide significant SNP associations (p<5.0x10-8) and array-wide significant SNP associations in Spencer et al., 2013 (p<3.1x10-7, Bonferroni correction for 161,098 tests) for these loci.

**When the index SNP was not genotyped on the MetaboChip, the proxy SNP in tight linkage disequilibrium (r2≥0.8 in 1000 Genomes pilot 1 CEU sample) with the lowest p-value in the African American sample was chosen to represent the index signal. If more than one SNP represented the same locus (within 500kb of each other) on the MetaboChip, only the SNPs r2<0.2 in ARIC African Americans (or HCHS/SOL Hispanic/Latinos when missing) were included in this table allowing preference for index SNPs, and in most cases SNPs from multiple citations. The decreasing and increasing alleles for proxies were assigned assuming that the risk index SNP would have a similar allele frequency (either minor or major) as the risk proxy SNP in 1000 Genomes EUR.

***Sample of African American women from ARIC (n=994) and WHI (n=866) with MetaboChip and age at natural menopause data. All other studies were of samples of European descent women.

****Modified random-effects trans-ethnic meta-analysis across three race/ethnic groups (African, Hispanic/Latina, and Asian Americans).

Significant SNP-associations (p<0.05/32 or 0.0016 for menarche; p<0.05/14 or 0.0036 for natural menopause) in bold. *Nominal SNP-associations in italics.*

All SNPs are oriented on positive strand and positions based on Build 37.
