# Supplementary material for: The genetic underpinnings of variation in ages at menarche and natural menopause among women from the multi-ethnic Population Architecture using Genomics and Epidemiology (PAGE) Study: A trans-ethnic meta-analysis
Source: PLoS One. 2018 Jul 25;13(7):e0200486. doi: 10.1371/journal.pone.0200486 (PMC6059436; doi:10.1371/journal.pone.0200486)
Supplement: S2 Table — (PDF) [file pone.0200486.s004.pdf]

Supplemental Table 2: Best marker SNPs at seven previously described age at menarche and natural menopause loci on the MetaboChip across multiple race/ethnic groups

GWAS Ref	Gene(s)	Chr	Start BP	Stop BP	number.Metabochip.SNPs	number SNPs r2<0.2*	Bonferroni P-value*	Index GWAS SNP	Position
	**MENARCHE**								
Elks *et al.*, 2010	*SEC16B*	1	177753776	177936525	767	177	2.82E-04	rs633715	177852580
Elks *et al.*. 2010	*TMEM18*	2	471136	719889	1126	299	1.67E-04	rs2947411	614168
Elks *et al.*. 2010	*TRIM66*	11	8394189	8707147	672	185	2.70E-04	rs4929923	8639200
Perry *et al.*, 2014	*BDNF*	11	27452706	27749725	691	202	2.48E-04	rs7103411	27700125
Perry *et al.*. 2014	*GPRC5B*	16	19704224	20019432	764	220	2.27E-04	rs12446632	19935389
Elks *et al.*, 2010	*FTO*	16	53539509	54185787	1817	557	8.98E-05	rs9939609	53820527
	**NATURAL MENOPAUSE**								
Stolk *et al.*, 2012	*FNDC4*	2	27386799	28670981	2702	792	6.31E-05	rs2303369	27715416

African American

Gene(s)	Top MetaboChip SNP	Position	Coded Minor Allele	Freq	Beta (years)	SE	P-value	P-value heterogeneity	N
SEC16B	rs75552107	177925677	G	0.158	0.127	0.04	6.85E-04	0.891	18599
TMEM18	rs75510653	552918	G	0.049	-0.160	0.05	3.92E-04	0.555	14815
TRIM66	rs10500703	8433864	A	0.062	-0.112	0.04	1.72E-03	0.743	20209
BDNF	rs113940328	27530168	C	0.053	0.140	0.04	**2.31E-04**	0.510	20209
GPRC5B	rs7199587	19847570	C	0.369	0.058	0.02	8.69E-04	0.911	20173
FTO	rs76299885	53950231	A	0.136	-0.119	0.04	3.48E-03	0.483	19545
FNDC4	rs114509965	27986156	G	0.023	-0.294	0.084	4.42E-04	0.0819	7297

Hispanic/Latina American

Gene(s)	Top MetaboChip SNP	Position	d Minor Allele	Freq	Beta (years)	SE	P-value	P-value heterogeneity	N
SEC16B	rs78368018	177777983	A	0.003	-0.825	0.21	**9.42E-05**	*0.038*	12787
TMEM18	rs79786235	663092	T	0.002	-0.936	0.28	9.04E-04	0.886	12384
TRIM66	rs35296353	8414229	T	0.016	0.233	0.08	4.94E-03	0.936	15347
BDNF	rs11030104	27684517	G	0.173	0.076	0.03	5.04E-03	0.851	15346
GPRC5B	rs72771066	19990613	G	0.156	-0.078	0.03	5.92E-03	0.296	15344
FTO	rs11237293(	53898024	A	0.017	0.325	0.09	5.28E-04	0.112	13527
FNDC4	rs2940800	28668615	T	0.084	0.32	0.103	1.92E-03	0.8014	5268

Asian American

Gene(s)	Top MetaboChip SNP	Position	Coded Minor Allele	Freq	Beta (years)	SE	P-value	P-value heterogeneity	N
SEC16B	rs7518576	177849963	T	0.459	0.081	0.03	1.19E-03	0.885	8265
TMEM18	rs6756518	651483	A	0.093	0.153	0.04	2.50E-04	0.901	8262
TRIM66	rs10743071	8395342	C	0.492	-0.073	0.02	2.37E-03	*0.001*	8273
BDNF	rs1491850	27749725	C	0.412	0.073	0.02	3.38E-03	0.933	8273
GPRC5B	rs72771069	19992816	T	0.069	-0.276	0.09	3.23E-03	0.595	8273
FTO	rs150356630	53830590	T	0.075	-0.198	0.05	1.43E-04	0.864	8273
FNDC4	rs11900743	28626860	A	0.011	-0.807	0.23	4.66E-04	0.2229	2346

Modified Random-Effects Trans-Ethnic**

Gene(s)	Top MetaboChip SNP	Position	Coded Minor Allele	Freq	Freq Range	Beta (years)	SE	Modified P-value	N
SEC16B	rs604388	177877979	C	0.43	0.28 - 0.63	-0.060	0.01	**3.39E-05**	31349
TMEM18	rs113407925	630868	A	0.10	0.05 - 0.14	0.093	0.02	2.69E-04	24173
TRIM66	rs11042029	8688125	A	0.50	0.35 - 0.70	-0.038	0.01	6.83E-03	37938
BDNF	rs4923463	27672500	G	0.18	0.06 - 0.43	0.068	0.02	**3.54E-05**	43824
GPRC5B	rs72771069	19992816	T	0.07	<0.01 - 0.12	-0.073	0.03	4.20E-03	43172
FTO	rs11642841	53845487	A	0.15	0.09 - 0.27	-0.073	0.02	**2.07E-05**	43797
FNDC4	rs9808353	28426725	A	0.29	0.01 - 0.62	-0.075	0.059	2.92E-04	16913

*Bonferroni correction for the number of SNPs r2<0.2 in region in MetaboChip data from the ARIC African Americans (n=1419 males, n=2332 females), using a 50-SNP window and shifting the window in each iteration by 5 SNPs.

**Strongest SNP marker in modified random-effects trans-ethnic meta-analysis across three race/ethnic groups (African, Hispanic/Latina, and Asian Americans).

Significant SNP-associations below specific Bonferroni p-value for a given locus in bold. *Nominal hetereogeneity p-values in italics.*

All SNPs are oriented on positive strand and positions based on Build 37.
