# Supplementary material for: The genetic underpinnings of variation in ages at menarche and natural menopause among women from the multi-ethnic Population Architecture using Genomics and Epidemiology (PAGE) Study: A trans-ethnic meta-analysis
Source: PLoS One. 2018 Jul 25;13(7):e0200486. doi: 10.1371/journal.pone.0200486 (PMC6059436; doi:10.1371/journal.pone.0200486)
Supplement: S3 Table — (PDF) [file pone.0200486.s005.pdf]

Supplemental Table 3: Descriptive statistics for the sample used in analysis of age at menarche

		African American (total n=20,209)						Hispanic/Latina American (total n=15,347)						Asian American (total n=8,273)			American Indian/Alaskan Native (total n=538)
		ARIC	EAGLE BioVU	CARDIA	MEC	BioME*	WHI*	HCHS/SOL	MEC	MEC SIGMA Diabetes Cases*	MEC SIGMA Diabetes Controls*	BioME*	WHI	MEC Hawaiian	MEC Japanese	WHI	WHI
Age at report (years)	N	2056	656	990	4410	373	11724	7027	859	910	910	512	5129	1364	3725	3184	538
	Mean (SD)	53 (5.7)	44 (16.5)	24 (3.9)	59 (9.0)	46 (13.5)	62 (7.1)	47 (13.5)	59 (7.5)	59 (7.0)	59 (7.0)	47 (14.1)	60 (6.8)	55 (7.7)	59 (8.5)	63 (7.5)	61 (7.4)
	Min, Max	44, 66	13, 99	17, 34	45, 77	18,86	50, 79	18, 76	45, 76	45, 76	45, 76	18, 89	49, 79	45, 75	45, 76	50, 79	50, 79
	Median	53	41	25	59	47	61	48	59	59	59	49	59	54	59	63	61
Birth Year	N	2056	656	990	4410	373	11724	7027	859	910	910	512	5129	1364	3725	3184	538
	Mean (SD)	1934 (5.8)	1969 (16.5)	1960 (3.9)	1933 (8.7)	1967 (13.5)	1934 (7.0)	1963 (13.5)	1933 (7.5)	1934 (7.1)	1934 (7.1)	1965 (14.1)	1935 (6.8)	1938 (7.9)	1934 (8.5)	1932 (7.4)	1934 (7.3)
	Min, Max	1921, 1945	1914, 2000	1950, 1968	1918, 1948	1927, 1995	1915, 1950***	1933, 1993	1918, 1948	1918, 1948	1918, 1948	1924, 1995	1915, 1950***	1918, 1953	1918, 1953	1915, 1950***	1915, 1950***
	Median	1935	1972	1960	1933	1966	1935	1962	1934	1935	1934	1964	1936	1939	1935	1933	1935
Age at Menarche (years)**	N	2056	656	990	4410	373	11724	7027	859	910	910	512	5129	1364	3725	3184	538
	Mean (SD)	12.88 (1.72)	12.56 (2.11)	12.51 (1.53)		12.48 (1.99)		12.57 (1.81)				12.33 (1.98)					
	Min, Max	8, 18	8, 20	8, 18		8, 19		8, 18				7, 18					
	Median	13	12	12		12		13				12					
	N<9 years	5	7	4		4		49				7					
	N>17 years	12	20	3		10		41				14					
Weight (kg)	N	2056	N/A	986	4308	373	11694	6961	853	910	910	512	5117	1362	3723	3181	535
	Mean (SD)	82.1 (18.0)		69.5 (18.4)	76.2 (17.1)	84.2 (25.3)	82.7 (18.8)	74.4 (16.8)	71.6 (14.2)	77.5 (16.4)	66.5 (11.1)	76.2 (19.0)	72.3 (16.0)	76.7 (18.6)	58.7 (11.1)	60.2 (13.0)	78.3 (18.5)
	Min, Max	37.3, 177.3		36.8, 158.8	42.6, 181.4	36,7, 206.8	32.0, 187.0	33.6, 217.7	39.5, 124.7	42.6, 167.8	43.1, 130.6	38.6, 158.8	38.2, 182.5	44.0, 204.1	35.4, 124.7	32.0, 163.8	42.0, 200.0
	Median	79.6		65.8	76.7	79.4	80.0	72.3	69.0	74.4	65.3	73.5	70.0	72.6	56.7	58.1	75.6
Height (cm)	N	2056	N/A	989	4358	373	11653	7017	856	910	910	512	5086	1360	3719	3171	532
	Mean (SD)	163.2 (6.2)		163.7 (7.2)	163.8 (6.7)	164.2 (7.7)	162.4 (6.6)	156.7 (6.4)	159.1 (6.1)	158.7 (6.0)	159.0 (6.2)	172.0 (7.0)	157.2 (6.2)	162.2 (6.5)	155.5 (5.7)	155.0 (5.9)	160.9 (7.1)
	Min, Max	125.0, 188.0		121.0, 188.0	134.6, 205.7	124.5, 188.0	90.0, 194.1	132.0, 189.0	121.9, 190.5	121.9, 190.5	124.5, 190.5	121.9, 180.3	93.5, 182.7	144.8, 210.8	124.5, 190.5	101.0, 177.9	106.3, 212.0
	Median	163.0		163.5	162.6	165.1	162.5	157.0	160	157.5	157.5	160.0	157.2	162.6	154.9	155.0	160.6
BMI (kg/m2)	N	2055	656	986	4284	373	11618	7010	851	910	910	512	5077	1359	3717	3170	528
	Mean (SD)	30.85 (6.63)	31.49 (8.66)	25.97 (6.47)	29.44(6.15)	31.09 (8.53)	31.18 (6.64)	30.26 (6.40)	28.34 (5.38)	30.80 (6.23)	26.39 (4.21)	30.18 (7.21)	29.07 (5.81)	29.14 (6.53)	24.32 (4.25)	24.91 (4.69)	29.97 (6.36)
	Min, Max	14.2, 65.91	13.47, 72.52	14.54, 53.53	14.80, 64.70	15.00, 70.00	11.53, 69.18	14.28, 70.35	17.03, 52.84	17.31, 67.82	15.36, 49.54	16.00, 62.00	15.54, 69.88	15.54, 68.57	12.16, 48.93	13.49, 65.61	16.61, 65.37
	Median	29.72	30.09	24.37	28.80	29.85	30.16	29.35	27.50	29.82	25.80	29.09	28.18	28.12	23.67	24.15	28.98
Obesity	N	2055	656	990	4284	373	11618	7010	851	910	910	512	5077	1359	3717	3170	528
	Yes, >=30.000kg/m^2	993	331	211	1690	179	5942	3177	272	448	155	231	1886	505	352	363	237
	No, <30.000kg/m^2	1062	325	779	2594	194	5676	3833	579	462	755	281	3191	854	3365	2807	291
	% Obese	48.3	50.5	21.3	39.4	48.0	51.1	45.3	32.0	49.2	17.0	45.1	37.1	37.2	9.5	11.5	44.9
Center/Region	N	2056	656	990	4410	373	11724	7027	859	910	910	512	5129	1364	3725	3184	538
		Forsyth: 236	Vanderbilt: 656	Birmingham: 274	Los Angeles/Haiwaii****: 4410	Mount Sinai: 373	Northeast: 2011	Bronx: 1885	Los Angeles/Haiwaii****: 859	Los Angeles: 910	Los Angeles: 910	Mount Sinai: 373	Northeast: 624	Haiwaii/Los Angeles****: 1364	Los Angeles: 3017	Northeast: 154	Northeast: 77
		Jackson: 1820		Chicago: 206			South: 5627	Chicago: 1510					South: 2112		Haiwaii: 708	South: 203	South: 128
				Minneapolis: 274			Midwest: 2771	Miami: 1947					Midwest: 204			Midwest: 137	Midwest: 57
				Oakland: 236			West: 1315	San Diego: 1685					West: 2189			West: 2690	West: 276

*Imputed MetaboChip SNP dosages used for MEC SIGMA, BioME, and part of WHI African American (n=6371) analytic samples. All other studies contributed only MetaboChip genotypes.

**For studies with continuous measures of age at menarche (ARIC, BioVU, CARDIA, HCHS/SOL, BioME).

***Minimum/maximum birth years for WHI rounded down/up to the nearest 5 year increment.

****Pooled across MEC study sites due to small sample from Haiwaii for African and Hispanic/Latina American women (n<5), and from Los Angeles for Haiwaiian women (n<5).

N/A= Not available
