# Supplementary material for: The genetic underpinnings of variation in ages at menarche and natural menopause among women from the multi-ethnic Population Architecture using Genomics and Epidemiology (PAGE) Study: A trans-ethnic meta-analysis
Source: PLoS One. 2018 Jul 25;13(7):e0200486. doi: 10.1371/journal.pone.0200486 (PMC6059436; doi:10.1371/journal.pone.0200486)
Supplement: S4 Table — (PDF) [file pone.0200486.s006.pdf]

Supplemental Table 4: Descriptive statistics for the sample used in analysis of age at natural menopause

		African American (total n=7,298)						Hispanic/Latina American (total n=5,271)							Asian American (total n=4,347)				American Indian/Alaskan Native (total n=184)
		ARIC	CARDIA	HyperGen	MEC	MESA	WHI*	HCHS/SOL	MEC	MEC SIGMA Diabetes Cases*	MEC SIGMA Diabetes Controls*	MESA	BioME*	WHI	MEC Hawaiian	MEC Japanese	MESA	WHI	WHI
Age at report (years)	N	569	150	189	1598	583	4209	1940	416	225	61	509	93	2027	567	1822	299	1659	184
	Mean (SD)	56 (4.8)	53 (2.3)	58 (8.7)	61 (7.9)	64 (8.9)	62 (7.1)	58 (6.5)	60 (6.7)	61 (7.0)	60 (7.0)	63 (9.4)	55 (4.1)	61 (6.8)	58 (7.1)	61 (7.4)	64 (9.3)	63 (7.4)	62 (7.7)
	Min, Max	45, 65	44,56	40, 79	45, 77	46,83	50, 79	42, 76	45, 75	45, 75	47, 75	45,84	45, 62	50, 79	45, 75	45, 76	45,84	50, 79	50, 78
	Median	56	54	57	62	65	62	58	60	60	61	63	55	60	57	61	65	63	61
Smoking Status	N	567	147	182	1583	583	4124	1938	400	213	60	509	20	1981	565	1813	299	1652	181
	Never	329	88	54	768	297	2078	1228	245	134	40	322	N/A	1303	272	1227	285	1189	95
	Ever	98	32	91	565	187	1566	392	108	59	15	125	N/A	555	180	435	9	393	68
	Current	140	27	37	250	99	480	318	47	20	5	62	20	123	113	151	5	70	18
	% Current	24.7	18.4	20.3	15.8	17.0	11.6	16.4	11.8	9.4	8.3	12.2	N/A	6.2	20.0	8.3	1.7	4.2	9.9
Birth Year	N	569	150	189	1598	583	4209	1940	416	225	61	509	93	2027	567	1822	299	1659	184
	Mean (SD)	1931 (4.9)	1957 (2.5)	1938 (8.7)	1931 (7.5)	1937 (8.9)	1934 (7.0)	1951 (6.6)	1933 (6.8)	1932 (7.0)	1933 (6.9)	1938 (9.4)	1958 (4.1)	1935 (6.7)	1935 (7.2)	1932 (7.3)	1937 (9.3)	1932 (7.4)	1934 (7.6)
	Min, Max	1921, 1944	1954, 1967	1918, 1956	1918, 1948	1918-1955	1915, 1950***	1933, 1969	1918, 1948	1918, 1948	1919, 1946	1917, 1956	1951, 1968	1915, 1950***	1918, 1953	1918, 1953	1917-1956	1915, 1950***	1915, 1950***
	Median	1931	1956	1940	1930	1936	1934	1952	1934	1933	1932	1938	1958	1936	1936	1932	1936	1932	1935
Age at Menopause (years)**	N	569	150	189	1598	583	4209	1940	416	225	61	509	93	2027	567	1822	299	1659	184
	Mean (SD)	48.3 (4.1)	48.8 (3.4)	47.2 (4.7)		49.0 (4.9)	50.0 (4.5)	48.5 (4.2)				48.6 (4.5)	49.4 (4.5)	49.6 (4.0)			49.7 (4.0)	50.2 (4.0)	49.3 (4.4)
	Min, Max	40, 60	40, 55	40,60		40, 60	40, 60	40, 60				40, 60	40, 59	40, 60			40, 60	40, 60	40, 60
	Median	49	49	47		49	50	49				49	50	50			50	50	50
Weight (kg)	N	569	149	189	1560	583	4196	1936	411	220	61	509	93	2022	567	1821	299	1659	182
	Mean (SD)	82.1 (18.1)	200.1 (50.0)	87.8 (19.6)	77.9 (16.9)	81.0 (17.3)	82.1 (18.8)	73.9 (15.6)	70.6 (13.2)	71.1 (13.7)	66.5 (12.4)	72.1 (14.3)	78.4 (20.5)	71.4 (15.3)	74.8 (17.8)	57.6 (10.1)	57.3 (9.4)	59.4 (12.6)	76.5 (16.4)
	Min, Max	39.5, 158.8	104.0, 376.5	45.4, 149.2	42.6, 181.4	39.5, 158.8	39.0, 171.5	34.5, 163.0	46.7, 124.7	47.6,124.7	46.7, 120.2	44.2, 133.2	43.5, 158.8	38.2, 165.0	44.0, 170.1	35.4, 113.4	35.9, 112.5	32.0, 163.8	46.5, 144.0
	Median	78.5	193.5	85.3	74.8	78.5	79.5	71.7	68.5	68.0	65.8	69.9	74.4	69.4	70.8	56.7	57.6	57.4	75.2
Height (cm)	N	569	149	189	1579	583	4177	1939	414	223	61	509	93	2012	566	1820	299	1651	182
	Mean (SD)	163.0 (6.12)	164.4 (6.6)	161.5 (19.6)	163.7 (6.5)	161.9 (6.6)	162.3 (6.9)	155.1 (6.2)	158.9 (6.5)	1589.0 (6.3)	157.8 (4.9)	154.8 (6.0)	159.1 (6.2)	157.1 (6.4)	162.2 (6.3)	155.1 (5.4)	154.9 (5.7)	154.8 (5.8)	161.4 (6.8)
	Min, Max	148.0, 185.0	150.0, 181.0	147.0, 176.0	139.7, 198.1	136.9, 183.3	95.0, 194.1	133.0, 176.2	121.9, 190.5	121.9, 177.8	147.3, 167.6	138.4, 178.8	101.6, 149.9	97.0, 177.1	144.8, 210.8	124.5, 188.0	137.8, 171.9	101.0, 177.9	144.6, 188.3
	Median	163.0	164.0	161.0	162.6	161.4	162.5	155.0	157.5	157.5	157.5	155.0	127.0	157.2	162.6	154.9	154.9	154.7	161.1
BMI (kg/m2)	N	569	149	189	1549	583	4158	1936	410	219	61	509	93	2009	566	1819	299	1651	181
	Mean (SD)	30.99 (6.79)	33.7 (8.6)	66.62 (7.07)	29.13 (6.11)	30.91 (6.46)	30.95 (6.67)	30.67 (5.88)	28.01 (5.02)	28.24 (5.33)	26.72 (4.54)	30.03 (5.49)	30.87 (7.24)	28.82 (5.74)	28.47 (6.31)	23.97 (3.92)	23.86 (3.51)	24.65 (4.59)	29.33 (6.03)
	Min, Max	16.25, 60.62	20.6, 65.3	16.26, 55.14	15.09, 64.70	15.87, 61.86	15.18, 69.18	15.62, 62.07	17.79, 52.07	17.79,52.07	18.88,44.19	18.3, 52.48	17.42, 58.23	15.54, 65.99	15.54, 58.86	12.16, 47.34	16.63, 43.67	13.49, 65.61	17.31, 49.81
	Median	29.70	32.10	32.47	28.21	30.04	30.02	29.94	27.40	27.18	26.58	29.30	29.99	27.97	27.33	23.46	23.77	23.87	28.58
Obesity	N	569	149	189	1549	583	4158	1936	410	219	61	509	93	2009	566	1819	299	1651	181
	Yes, >=30.000kg/m^2	277	94	119	582	302	2084	953	126	67	12	257	51	714	185	135	16	165	74
	No, <30.000kg/m^2	292	55	70	967	281	2074	983	284	152	49	252	42	1295	381	1684	283	1486	107
	% Obese	48.7	63.1	63.0	37.6	51.8	50.1	49.2	30.7	30.6	19.7	50.5	54.8	35.5	32.7	7.4	5.4	10.0	40.9
Center/Region	N	569	150	189	1598	583	4209	1940	416	225	61	509	93	2027	567	1822	299	1659	184
		Forsyth: 91	Birmingham: 29	Birmingham: 145	Los Angeles/Haiwaii****: 1598	WFU: 146	Northeast: 915	Bronx: 487	Los Angeles: 416	Los Angeles: 225	Los Angeles: 61	WFU: 0	Mount Sinai: 93	Northeast: 374	Haiwaii/Los Angeles****: 567	Haiwaii: 1416	WFU: 0	Northwest: 99	Northwest: 29
		Jackson: 478	Chicago: 37	Forsyth: 44		COL: 111	South: 1864	Chicago: 399				COL: 171		South: 836		Los Angeles: 406	JFU: 0	South: 106	South: 46
			Minneapolis: 30			JHU: 165	Midwest: 977	Miami: 650				JHU: 0		Midwest: 80			UMN: 0	Midwest: 89	Midwest: 18
			Oakland: 54			UMN: 0	West: 453	San Diego: 404				UMN: 160		West: 737			NWU/COL****: 112	West: 1365	West: 91
						NWU: 111						NWU: 0					UCLA: 187		
						UCLA: 50						UCLA: 178							

*Imputed MetaboChip SNP dosages used for MEC SIGMA, BioME, and part of WHI African American (n=2276) analytic samples. All other studies contributed only MetaboChip genotypes.

**For studies with continuous measures of age at menopause (ARIC, CARDIA, HCHS/SOL, HyperGen, MESA, BioME, WHI).

***Minimum/maximum birth years for WHI rounded down/up to the nearest 5 year increment.

****Pooled across MEC study sites due to small sample from Haiwaii for African and Hispanic/Latina American women (n<5), and from Los Angeles for Haiwaiian women (n<5). MESA Asian American women from Columbia University in New York City were pooled due to small sample size (n<5).

N/A= Not available
