# Supplementary material for: The genetic underpinnings of variation in ages at menarche and natural menopause among women from the multi-ethnic Population Architecture using Genomics and Epidemiology (PAGE) Study: A trans-ethnic meta-analysis
Source: PLoS One. 2018 Jul 25;13(7):e0200486. doi: 10.1371/journal.pone.0200486 (PMC6059436; doi:10.1371/journal.pone.0200486)
Supplement: S5 Table — (PDF) [file pone.0200486.s007.pdf]

Supplemental Table 5. Using the set of SNPs in high LD (r2≥0.8)* with European index SNP in African, Hispanic, and Asian American populations to narrow of the region**

				Number of SNPs in LD (r2≥0.8)*				European			African			Hispanic/Latino			East Asian			% Reduction**
Gene	Index GWAS SNP	Chr	Position	European	African	Hispanic	Asian	Start pos	End pos	Range	Start pos	End pos	Range	Start pos	End pos	Range	Start pos	End pos	Range	
**MENARCHE**																				
SEC16B	rs633715	1	177852580	54	10	12	16	177793822	177913519	119697	177855517	177882786	27269	177855517	177881651	26134	177855517	177889480	33963	78
TMEM18	rs2947411	2	614168	216	1	1	35	600575	653874	53299	615627	615627	0	615627	615627	0	613044	635864	22820	100
TRIM66	rs4929923	11	8639200	28	3	33	46	8473707	8694073	220366	8636105	8683639	47534	8636105	8694073	57968	8636105	8694073	57968	78
BDNF	rs7103411	11	27700125	42	5	29	38	27634373	27742447	108074	27703188	27728539	25351	27666984	27742447	75463	27643725	27749725	106000	77
GPRC5B	rs12446632	16	19935389	70	7	10	31	19707417	19977733	270316	19931360	19942331	10971	19931114	19942331	11217	19717915	19994023	276108	96
FTO	rs9939609	16	53820527	88	25	37	39	53799507	53845169	45662	53816275	53822651	6376	53810546	53822651	12105	53809247	53822651	13404	86
**NATURAL MENOPAUSE**																				
FNDC4	rs2303369	2	27715416	142	20	43	131	27548038	27752692	204654	27566989	27714526	147537	27566989	27740404	173415	27527246	27738854	211608	28

*Other than the index SNP. 1000 Genomes phase 1 samples used to capture European (CEU), African (YRI), Hispanic/Latino (MXL, PUR, CLM), and East Asian (JPT) linkage disequilibirum patterns.

**% Reduction is defined by: 1- (Population with minimum range)/ (Population with maximum range) x 100%

All SNPs are oriented on positive strand and positions based on Build 37.
