# Supplementary material for: The genetic underpinnings of variation in ages at menarche and natural menopause among women from the multi-ethnic Population Architecture using Genomics and Epidemiology (PAGE) Study: A trans-ethnic meta-analysis
Source: PLoS One. 2018 Jul 25;13(7):e0200486. doi: 10.1371/journal.pone.0200486 (PMC6059436; doi:10.1371/journal.pone.0200486)
Supplement: S2 Fig — (PDF) [file pone.0200486.s009.pdf]

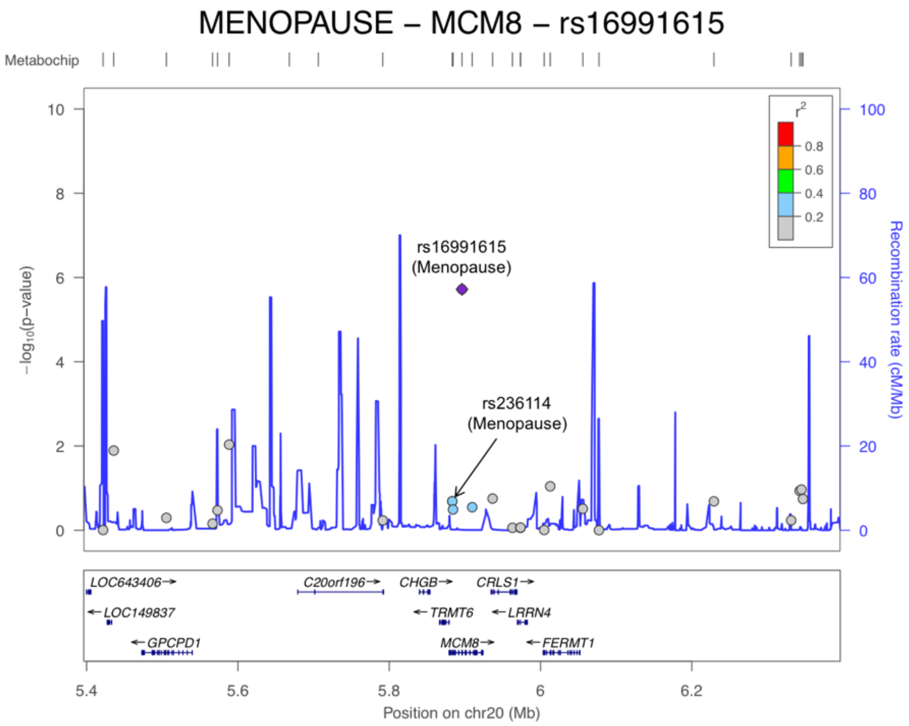

Supplemental Figure 2: Regional plot for trans-ethnic Bonferroni-significant association signal between *MCM8* and ANM using a modified random-effects trans-ethnic meta-analysis of more than 16,000 women
