# Supplementary material for: The genetic underpinnings of variation in ages at menarche and natural menopause among women from the multi-ethnic Population Architecture using Genomics and Epidemiology (PAGE) Study: A trans-ethnic meta-analysis
Source: PLoS One. 2018 Jul 25;13(7):e0200486. doi: 10.1371/journal.pone.0200486 (PMC6059436; doi:10.1371/journal.pone.0200486)
Supplement: S3 Fig — (PDF) [file pone.0200486.s010.pdf]

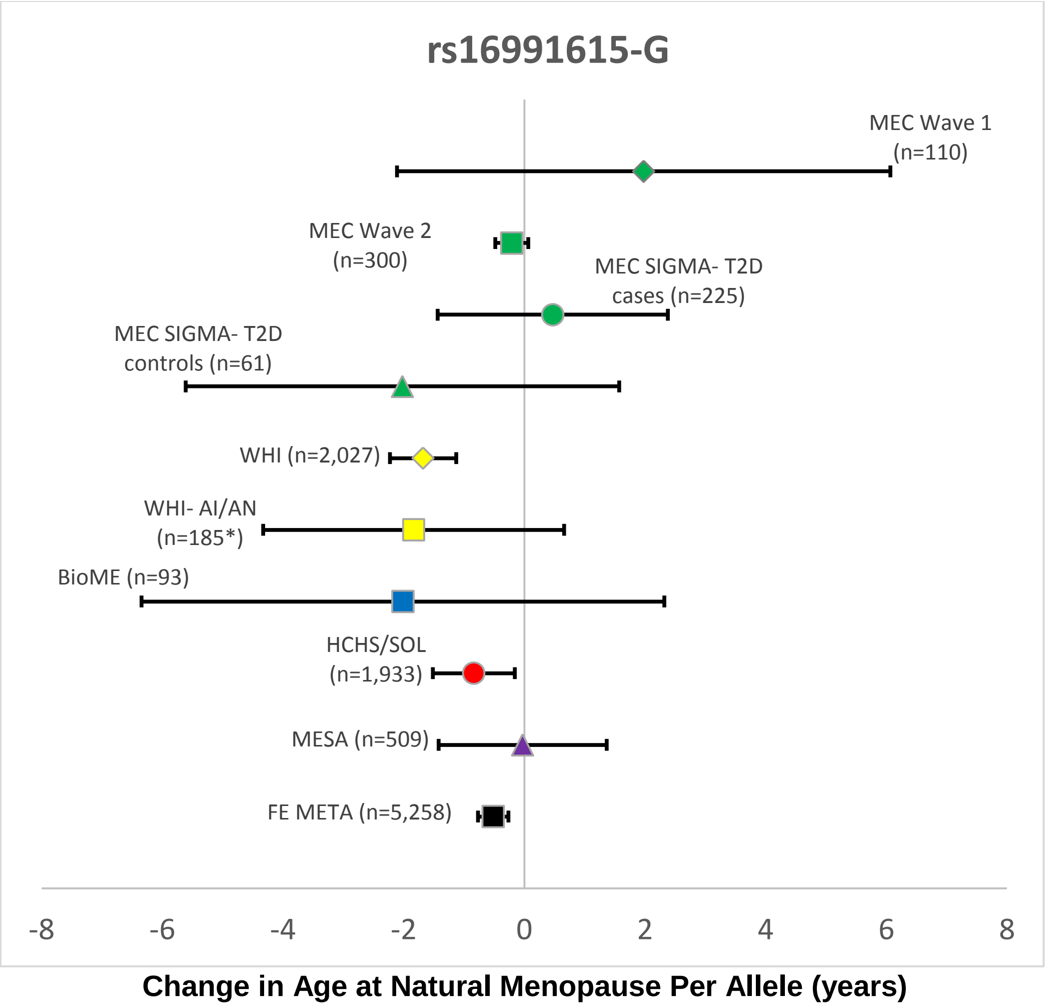

Supplemental Figure 3: Forest plot of effect (p-value of heterogeneity= $4x10^{-4}$) in the fixed-effect meta-analysis (FE META) across eight study samples [Multiethnic Cohort Study=MEC, MEC-Slim Initiative in Genomic Medicine for the Americas Type 2 Diabetes Consortium=MEC SIGMA, Women's Health Initiative=WHI, WHI American Indian/Alaskan Native=WHI AI/AN (*not included in Hispanic/Latina fixed-effect meta-analysis), Mount Sinai School of Medicine BioBank=BioME, Hispanic Community Health Study/Study of Latinos=HCHS/SOL, Multi-Ethnic Study of Atherosclerosis=MESA) of 5,258 Hispanic/Latinas at an index SNP at *MCM8* (rs16991615) with ANM
