# Supplementary material for: The genetic underpinnings of variation in ages at menarche and natural menopause among women from the multi-ethnic Population Architecture using Genomics and Epidemiology (PAGE) Study: A trans-ethnic meta-analysis
Source: PLoS One. 2018 Jul 25;13(7):e0200486. doi: 10.1371/journal.pone.0200486 (PMC6059436; doi:10.1371/journal.pone.0200486)
Supplement: S4 Fig — (PDF) [file pone.0200486.s011.pdf]

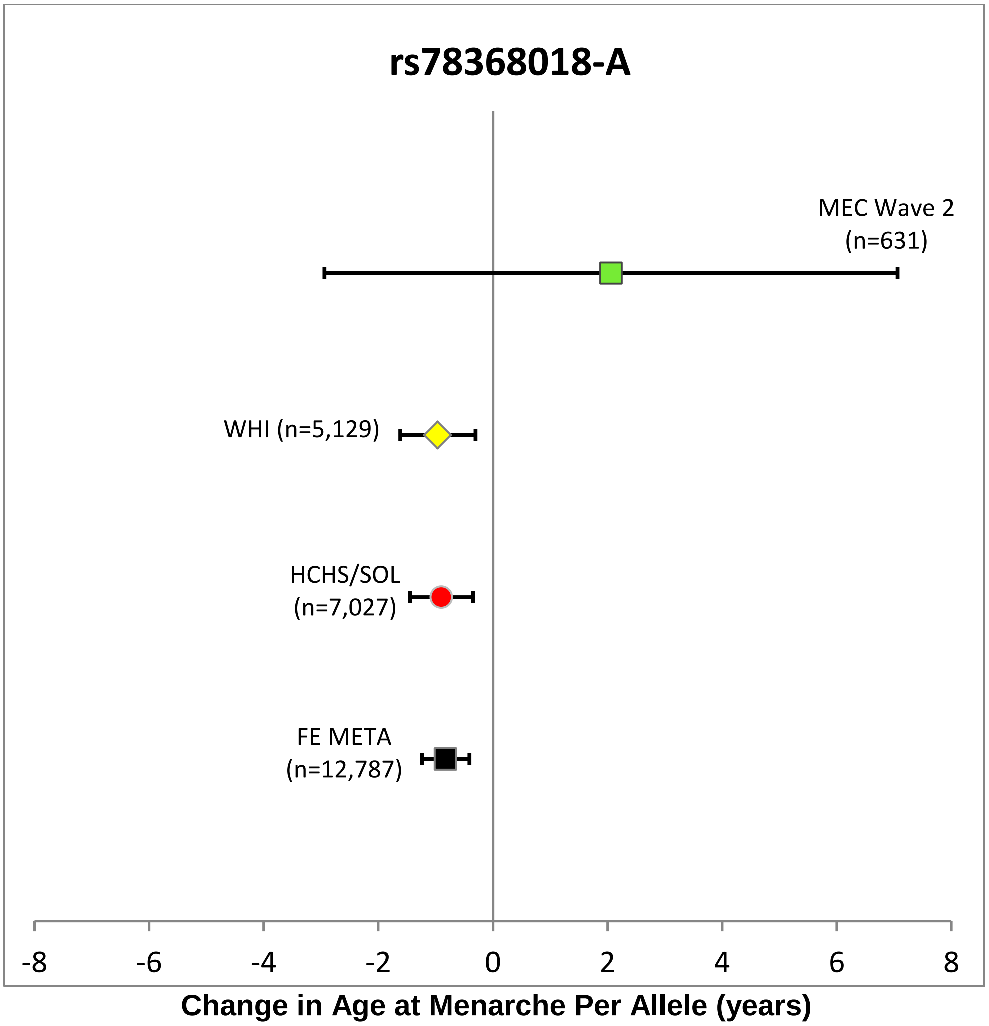

Supplemental Figure 4: Forest plot of effect heterogeneity (p-value of heterogeneity= 0.04) across three studies (Multiethnic Cohort Study=MEC, Women's Health Initiative=WHI, Hispanic Community Health Study/Study of Latinos=HCHS/SOL) of 12,787 Hispanic/Latinas at the best-marker fixed-effect meta-analysis (FE-META) at *SEC16B* (rs78368018) with AAM.
