# Supplementary material for: The genetic underpinnings of variation in ages at menarche and natural menopause among women from the multi-ethnic Population Architecture using Genomics and Epidemiology (PAGE) Study: A trans-ethnic meta-analysis
Source: PLoS One. 2018 Jul 25;13(7):e0200486. doi: 10.1371/journal.pone.0200486 (PMC6059436; doi:10.1371/journal.pone.0200486)
Supplement: S6 Fig — (PDF) [file pone.0200486.s013.pdf]

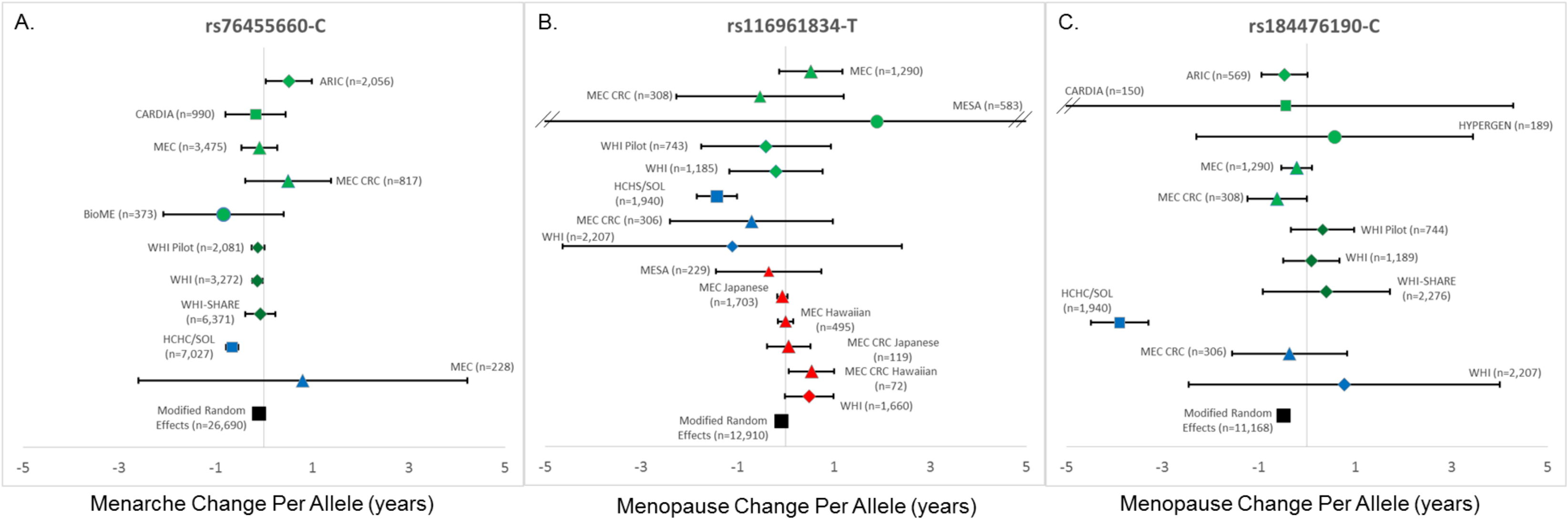

Supplemental Figure 6: Forest plots of three novel array-wide significant modified random-effect estimates (p-values$<4x10^{-8}$; shown by the black box) and the contributing race/ethnic (African American in green; Hispanic/Latina in blue; Asian American in red) and study-specific effect estimates and their 95% confidence intervals (with double bars indicating full range not shown
