# Supplementary material for: The genetic underpinnings of variation in ages at menarche and natural menopause among women from the multi-ethnic Population Architecture using Genomics and Epidemiology (PAGE) Study: A trans-ethnic meta-analysis
Source: PLoS One. 2018 Jul 25;13(7):e0200486. doi: 10.1371/journal.pone.0200486 (PMC6059436; doi:10.1371/journal.pone.0200486)
Supplement: S7 Fig — (PDF) [file pone.0200486.s014.pdf]

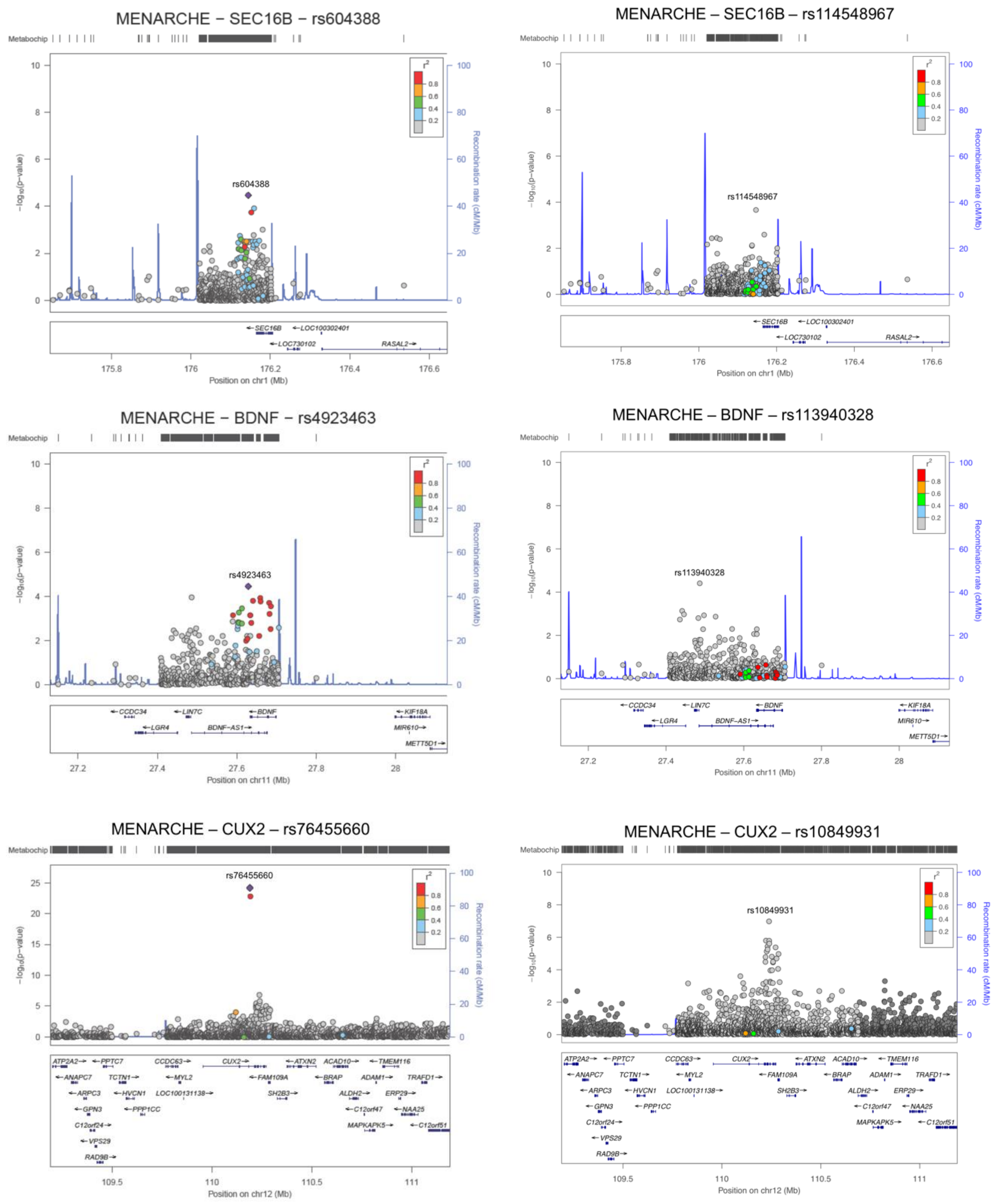

Supplemental Figure 7: Regional plots of unconditional findings (left; r^2 based off of significant lead unconditional SNP) and approximate conditional findings after accounting for top SNPs in the region (right; r^2 based off of unconditional lead SNP, noting significant lead conditional SNP) with age at menarche at *SEC16B, BDNF*, and *CUX2* in more than 31,000 women
