# Supplementary material for: The genetic underpinnings of variation in ages at menarche and natural menopause among women from the multi-ethnic Population Architecture using Genomics and Epidemiology (PAGE) Study: A trans-ethnic meta-analysis
Source: PLoS One. 2018 Jul 25;13(7):e0200486. doi: 10.1371/journal.pone.0200486 (PMC6059436; doi:10.1371/journal.pone.0200486)
Supplement: S5 Fig — (PDF) [file pone.0200486.s012.pdf]

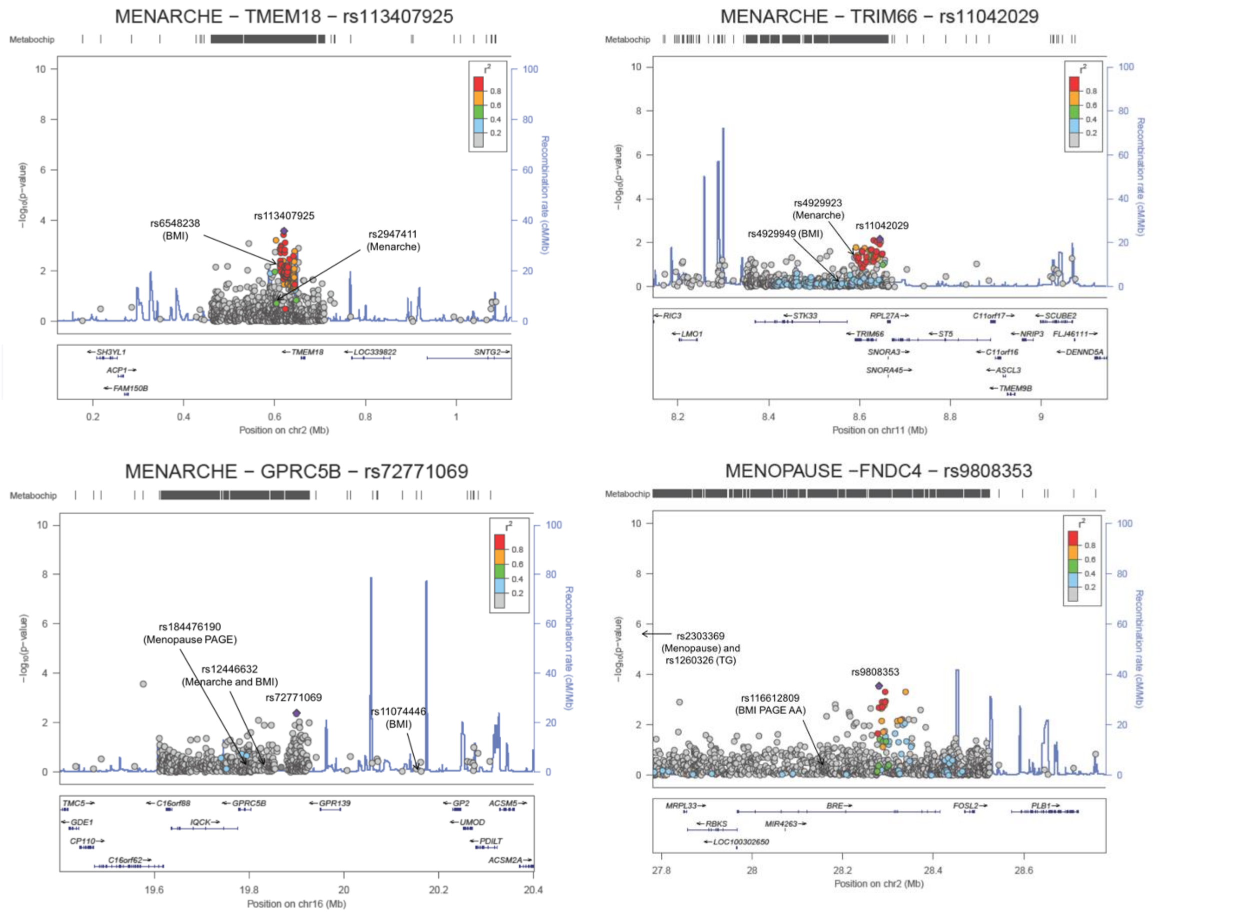

Supplemental Figure 5: Regional plots of non-significant modified random-effects trans-ethnic associations with dense-genotyped reproductive loci on the MetaboChip (AAM: *TMEM18, TRIM66, GPRC5B*; ANM: *FNDC4*) in up to 43,172 women with age at menarche and 16,913 women with age at natural menopause, showing the SNPs associated with body mass index (BMI) and triglycerides (TG) in previous studies, including the PAGE Study African American (AA) subsample, as well as another SNP that was associated with the other reproductive trait in this sample
